# A Phyto-mycotherapeutic Supplement, Namely *Ganostile*, as Effective Adjuvant in Brain Cancer Management: An In Vitro Study Using U251 Human Glioblastoma Cell Line

**DOI:** 10.3390/ijms25116204

**Published:** 2024-06-05

**Authors:** Ludovica Gaiaschi, Fabrizio De Luca, Elisa Roda, Beatrice Ferrari, Claudio Casali, Chiara Rita Inguscio, Federica Gola, Enrico Pelloni, Elena Savino, Mauro Ravera, Paola Rossi, Maria Grazia Bottone

**Affiliations:** 1 Department of Biology and Biotechnology “L. Spallanzani”, University of Pavia, 27100 Pavia, Italy; ludovica.gaiaschi@unipv.it (L.G.); beatrice.ferrari01@universitadipavia.it (B.F.); claudio.casali@unipv.it (C.C.); chiara.inguscio01@universitadipavia.it (C.R.I.); federica.gola01@universitadipavia.it (F.G.); enrico.pelloni01@universitadipavia.it (E.P.); paola.rossi@unipv.it (P.R.); mariagrazia.bottone@unipv.it (M.G.B.); 2 Laboratory of Clinical & Experimental Toxicology, Pavia Poison Centre, National Toxicology Information Centre, Toxicology Unit, Istituti Clinici Scientifici Maugeri IRCCS, 27100 Pavia, Italy; elisa.roda@icsmaugeri.it; 3 Department of Earth and Environmental Sciences (DSTA), University of Pavia, Via Ferrata 1, 27100 Pavia, Italy; elena.savino@unipv.it; 4 Department of Sciences and Technological Innovation (DiSIT), University of Piemonte Orientale “A. Avogadro”, Viale Teresa Michel 11, 15121 Alessandria, Italy; mauro.ravera@uniupo.it

**Keywords:** glioblastoma, phytotherapy, mitochondrial disfunction, oxidative stress, ferroptosis, drug resistance

## Abstract

The current standard oncotherapy for glioblastoma is limited by several adverse side effects, leading to a short-term patient survival rate paralleled by a worsening quality of life (QoL). Recently, Complementary and Integrative Medicine’s (CIM) innovative approaches have shown positive impacts in terms of better response to treatment, side effect reduction, and QoL improvement. In particular, promising potential in cancer therapy has been found in compounds coming from phyto- and mycotherapy. The objective of this study was to demonstrate the beneficial effects of a new phyto-mycotherapy supplement, named *Ganostile*, in the human glioblastoma cell line U251, in combination with chemotherapeutic agents, i.e., Cisplatin and a new platinum-based prodrug. Choosing a supplement dosage that mimicked oral supplementation in humans (about 1 g/day), through in vitro assays, microscopy, and cytometric analysis, it has emerged that the cells, after 48hr continuous exposure to *Ganostile* in combination with the chemical compounds, showed a higher mortality and a lower proliferation rate than the samples subjected to the different treatments administered individually. In conclusion, our data support the use of *Ganostile* in integrative oncology protocols as a promising adjuvant able to amplify conventional and new drug effects and also reducing resistance mechanisms often observed in brain tumors.

## 1. Introduction

Glioblastoma is the most common and aggressive primary brain neoplasm. The current treatments are surgery resection, chemotherapy, and radiation therapy, but the development of relapses and resistance is almost inevitable [1].

In recent years, the therapeutic power of medicinal mushrooms has become very popular in alternative and integrative medicine and, to date, the use of mycotherapy could represent a new strategy to support standard therapies and consequently improve the treatment of different tumors. Thanks to the several benefits that these natural substances bring to the whole organism, the therapy would not be limited only to the resolution of the symptoms but would significantly improve the quality of life of the patients themselves [2]. In mycotherapy, among the most known and studied mushrooms for their curative properties are *Ganoderma lucidum*, *Hericium erinaceus*, and *Ophiocordyceps sinensis*.

*Ganoderma lucidum*, also known as Ling Zhi in China or Reishi in Japan, is a fungal species belonging to Basidiomycota, perennial mushrooms, saprophytes that grow and develop on deciduous oak and chestnut trees [3]. For over 2000 years, *G. lucidum* has been used in traditional oriental medicine thanks to its multiple beneficial effects as a therapeutic agent for health and longevity, showing great efficacy in the treatment of many diseases, including cancer [4]. Several classes of bioactive substances have been isolated and identified from *G. lucidum*, such as triterpenoids, polysaccharides, nucleosides, sterols, and alkaloids. Among all the various bioactive compounds previously reported, triterpenoids and polysaccharides represent the main components responsible for anticancer activity [5,6]. The potential role of *G. lucidum* polysaccharides (GLPs) is to activate T and B lymphocytes, macrophages, dendritic cells, and natural killer cells, promoting lymphocytes proliferation, phagocytosis, and cytokines production [7]. Moreover, data suggest that GLPs suppress tumorigenesis or inhibit tumor growth through a direct cytotoxic effect and antiangiogenic activity [8]. Indeed, in vitro and in vivo studies have shown that high-molecular-weight polysaccharide components, such as β-D-glucans, have antitumor activity thanks to their immunomodulatory and antiproliferative effects, exerting proapoptotic, antimetastatic, and antiangiogenic actions [9]. On the other hand, triterpenoids also display antiproliferative effects, antiangiogenic activity [10], and anti-inflammatory and antimetastatic actions [11,12].

The promising effects of *G. lucidum* on both proliferation and migration of glioblastoma (GBM) cell models have already been highlighted by previous studies [13], which, however, have left the mechanisms of action of the mycotherapeutic substance unexplored. In addition, *G. lucidum* can be associated with other types of myco-phytotherapy, e.g., *Agaricus blazei*, *Ophiocordyceps sinensis*, *Grifola frondosa*, and *Lentinula edodes*, in order to improve the conditions of oxidative stress present in the body, fundamental as a method of preventing other diseases [14,15,16].

Here we used, as a source of *G. lucidum*, the *Ganostile* supplement (Miconet s.r.l.) that also contains extract from *Eleutherococcus senticosus*, *Echinacea purpurea*, and *Astragalus membranaceus*. *E. senticosus*, as *E. purpurea*, is a species of the *Araliaceae* family [17]; it contains flavonoids, organic acids, phenols, triterpenoid saponins, lignans, coumarins, and polysaccharides and is known for its positive effects on neurasthenia, hypertension, immune regulation, cerebrovascular diseases, and ischemic heart diseases, and it also contains phenolic compounds such as caffeic acid and chlorogenic acid, which have antioxidant properties [18]. *Echinacea purpurea* is characterized by bioactive components with antiviral, anti-inflammatory, bacteriostatic, and immunoregulatory activities. Among the plant’s compounds, some polysaccharides have an antioxidant action [19]. *Astragalus membranaceus*, from the *Fabaceae* family, is also mainly known for its immunomodulating, antioxidant, anti-inflammatory, and anticancer properties. Concerning antineoplastic activity, combined treatment with chemotherapy and *A. membranaceus* has been shown to improve drug-induced toxicity in gastrointestinal cancers [20].

This study aimed to evaluate the effects on human glioblastoma U251 cells induced by the natural-based supplement *Ganostile* (Miconet s.r.l). Parallelly, the combination of *Ganostile* with Cisplatin (CisPt) or with a new platinum-based prodrug ((OC-6-44)-acetatodiamminedichlorido(2-(2-propynyl)octanoato)platinum(IV)), named Pt(IV)Ac-POA (Pt-POA), already tested on different tumor cell types [21,22,23], has been tested on that same tumor cell line. Using cell viability assay, flow cytometric, immunocytochemical, and electron microscopy techniques, the effects induced following continuous exposure to the supplement and combined treatments were evaluated, highlighting the possible effects at subcellular levels and the antiproliferative activity, as well as modulations of oxidative stress and cell death mechanisms.

## 2. Results

**Figure 1:**
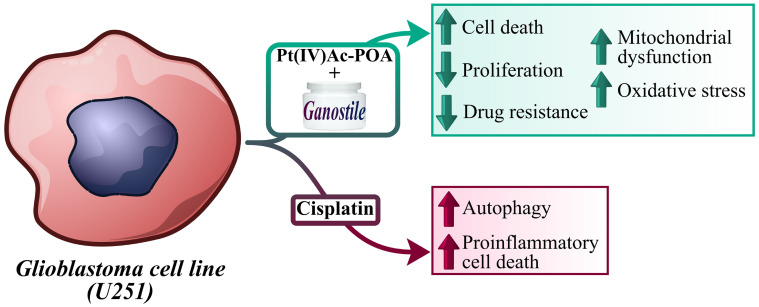
Graphic illustration underlining the main results.

The key outcomes of the present investigation are emphasized in Figure 1.

### 2.1. Determination of the Concentration of Ganostile

**Figure 2:**
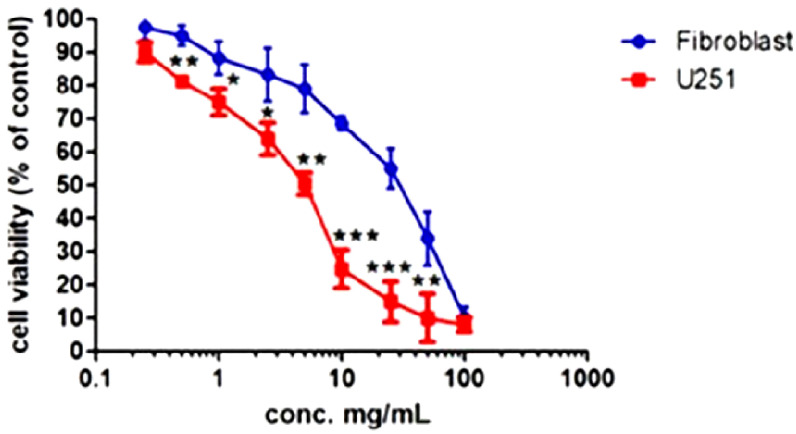
MTS viability assay. The cytotoxicity of the *Ganostile* was evaluated in cultured U251 and primary human fibroblast cells, concentrations from 0.25 to 100 mg/mL were used for 48hr. Cell viability was compared to untreated controls, cultured in the presence of the only basic growing medium. The experiments were performed two times with three technical replicates for each treatment. Data are expressed as mean ± SD referring to the control. *p* value: (*) < 0.05; (**) < 0.01 and (***) < 0.001.

By MTS assay, a *Ganostile* concentration of 5 mg/mL (G-5 mg/mL) was found to be the half maximal inhibitory concentration, causing a significant decrease (about 50%) in the number of living cells (Figure 2). As a comparison, primary culture of human fibroblasts was used and a 50% reduction (55.2 ± 6.1%) in cell viability compared to the untreated sample was induced by supplement at 25 mg/mL, a concentration 5-fold higher than that obtained for the U251 treatment.

**Figure 3:**
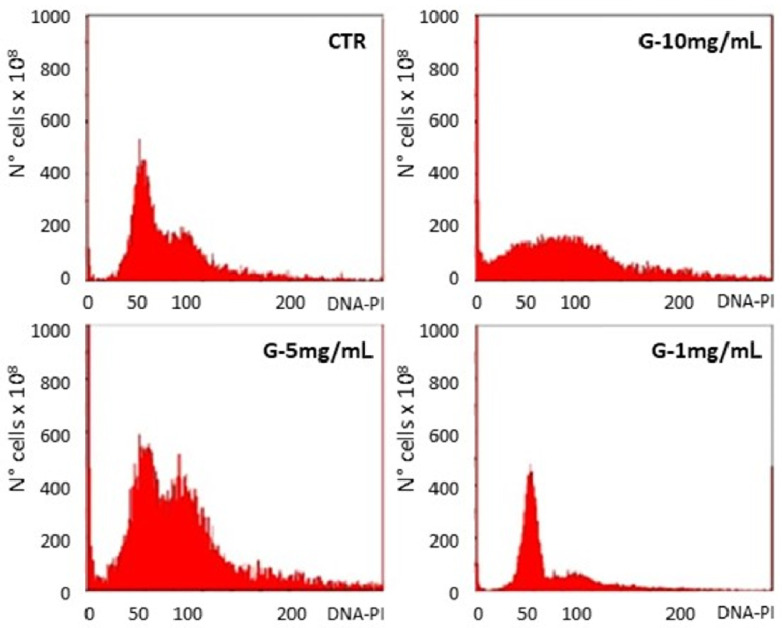
Cytofluorimetric analysis of DNA content in U251 cells after PI staining in control conditions (CTR) and after treatment with *Ganostile* at different concentrations: 10 mg/mL, 5 mg/mL, and 1 mg/mL.

Therefore, narrowing down the *Ganostile* dose range, three concentrations (1, 5, and 10 mg/mL), including the IC_50_ identified by MTS assay and those immediately higher and lower, were selected to assess possible *Ganostile*-induced changes/alterations in the U251 cell cycle. Briefly, after 48hr-CT with the supplement, cytofluorimetric analysis of U251 cell DNA content was performed (Figure 3).

Compared to the control, in which 63.01% of the cells were found in the G1 phase, 14.06% in M phase, and 22.93% in G2 phase, at the concentration of 10 mg/mL, the cells no longer showed a normal distribution in the various phases of the cycle but there was a complete alteration in the cell cycle (G1 phase 34.71%, M phase 31.33%, and G2 phase 33.96). In contrast, no major changes were given by the usage of *Ganostile* 1 mg/mL; indeed, a pattern of cell distribution very similar to the control was notable (G1 phase 72.65%, M phase 12.70%, and G2 phase 14.65), meaning that, at this concentration, cells were not damaged considerably.

Finally, at a concentration of 5 mg/mL, there was a more evident sub-G1 peak than in the control; note that 50.20% of cells were in the G1 phase and 38.56% in the M phase, while 11.24% were found in phase G2, highlighting a blockage of the cells in the G2/M phase of the cell cycle. Based on these data, the concentration of 5 mg/mL was chosen for subsequent treatments and investigations.

### 2.2. Proliferation Pathway Evaluation

**Figure 4:**
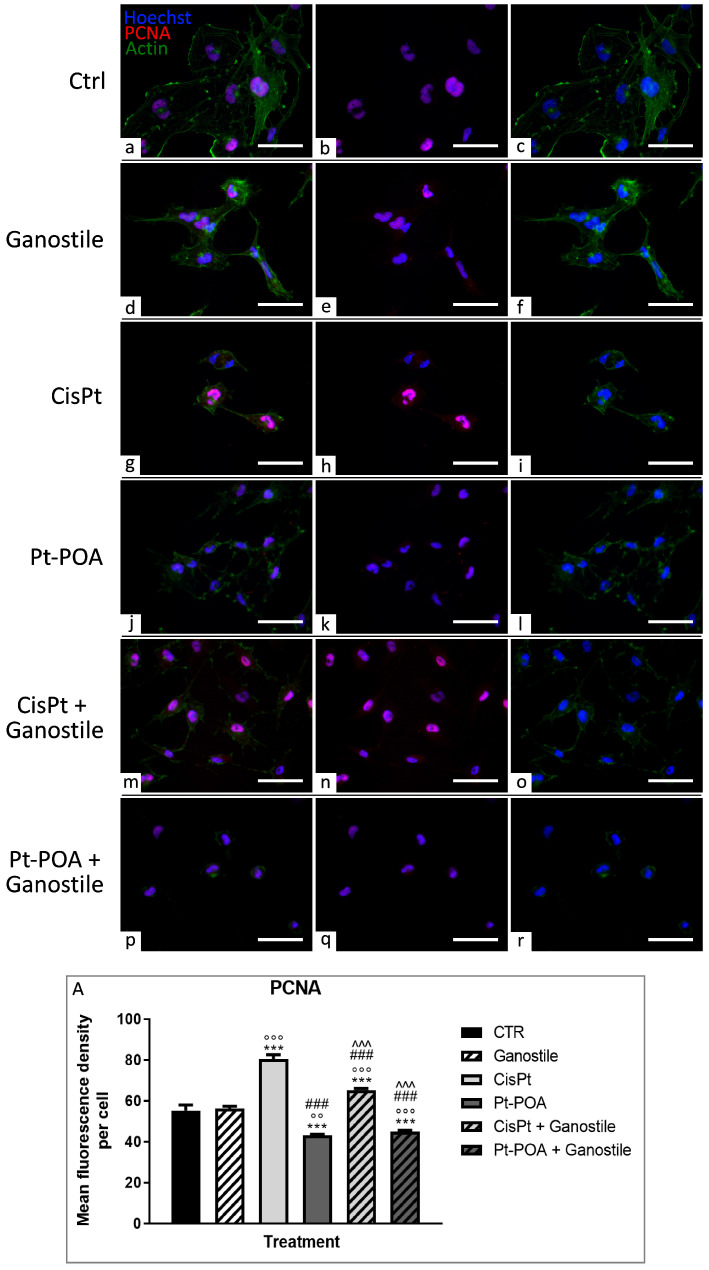
Double immunolabeling for PCNA (in red) and actin (in green) in controls (**a**–**c**) and differently treated U251 cells, i.e., after 48hr-CT using *Ganostile* 5 mg/mL (**d**–**f**), Cisplatin 40 μM (**g**–**i**), Pt(IV)Ac-POA 10 μM (**j**–**l**), Cisplatin + *Ganostile* (**m**–**o**), or Pt(IV)Ac-POA + *Ganostile* (**p**–**r**). DNA was stained with Hoechst 33258 (blue fluorescence). Magnification 40×, scale bar: 50 μm. (Panel **A**) Histogram showing the PCNA mean fluorescence intensity per cell (OD) in control and differently treated cells. * control vs. each experimental condition; ° *Ganostile* vs. other treatments; # Cisplatin vs. any combined treatment; ^ Pt(IV)Ac-POA vs. each combined treatment.

To further assess cell proliferation and the DNA damage repair mechanism, proliferating cell nuclear antigen (PCNA) immunolabeling, coupled with actin staining, was evaluated (Figure 4).

A strong nuclear labeling of PCNA was observed in the control (a–c) condition (55.30 ± 8.79), suggesting the presence of cycling cells; note the presence of several mitotic cells (b). Furthermore, in the control group, the actin cytoskeleton showed an astrocytic-like structure with numerous pseudopodia (a). After treatment with *Ganostile* 5 mg/mL (d–f), no acute effect on nuclear PCNA fluorescence was detected (56.23 ± 3.83). Nevertheless, PCNA fluorescence spread out of the nucleus to indicate a rupture of the nuclear membrane and a process of cytoplasmic degradation (e).

The treatments with Cisplatin alone (g–i) and in combination with *Ganostile* (m–o) showed a rise in PCNA levels (80.35 ± 7.31 and 65.29 ± 2.49, respectively). In contrast, a reduction in PCNA immunofluorescence OD was observed after the administration of Pt(IV)Ac-POA (j–l) and its combination (p–r) (43.19 ± 1.48 and 44.99 ± 2.04 for Pt(IV)Ac-POA and *Ganostile* + Pt(IV)Ac-POA, respectively). Moreover, treatments with Pt(IV)Ac-POA lead to a significant immunopositive decrease in the investigated proliferative marker compared to the control, as demonstrated by the presence of several PCNA immunonegative nuclei (k,q). Furthermore, cellular cytoskeleton and nuclear structures completely lost their physiological organization, i.e., several cells showed (i) pycnotic nuclei, characterized by dense and fragmented chromatin, and (ii) aggregated fluorescence spots of actin.

Specifically, the samples treated with *Ganostile* + Pt(IV)Ac-POA showed cells with a particularly small size compared to those of the other samples, characterized by actin cytoskeleton (r) completely collapsing (Figure 4, Panel A).

### 2.3. Oxidative Stress Pathway Evaluation

**Figure 5:**
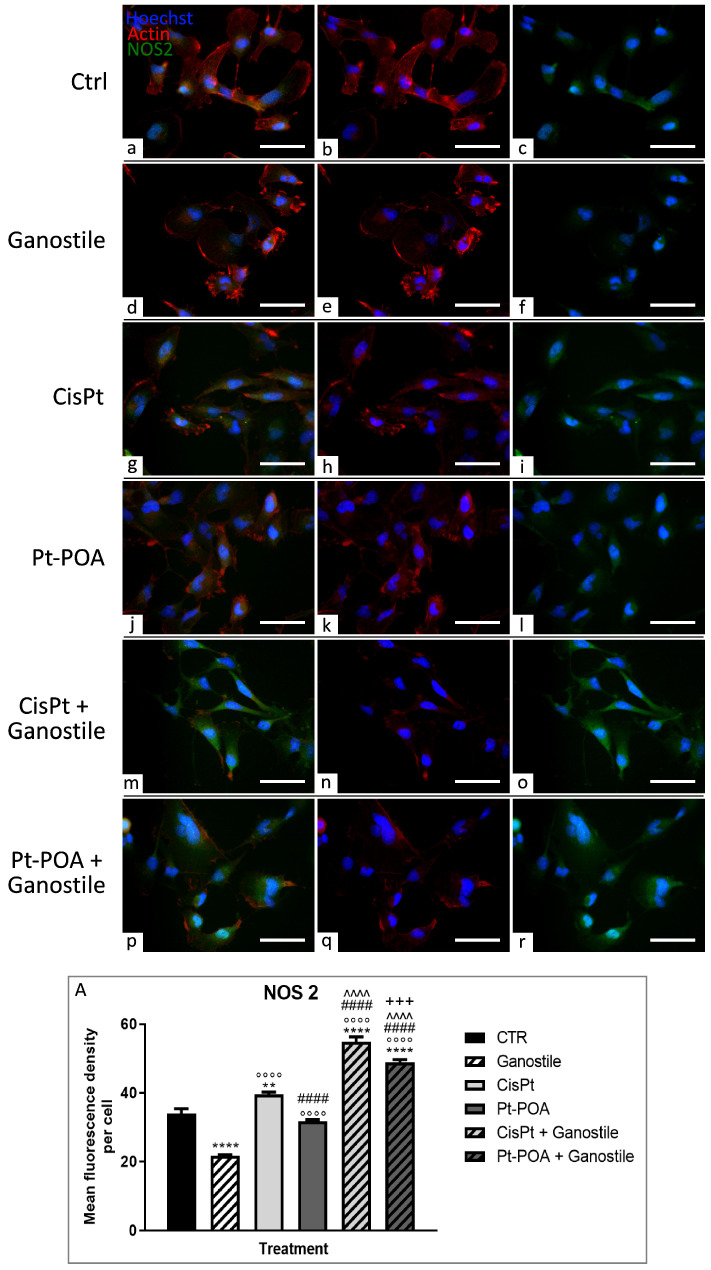
Double immunolabeling for NOS (in green) and actin (in red) in controls (**a**–**c**) and differently treated U251 cells, i.e., after 48hr-CT using *Ganostile* 5 mg/mL (**d**–**f**), Cisplatin 40 μM (**g**–**i**), Pt(IV)Ac-POA 10 μM (**j**–**l**), Cisplatin + *Ganostile* (**m**–**o**), or Pt(IV)Ac-POA + *Ganostile* (**p**–**r**). DNA was stained with Hoechst 33258 (blue fluorescence). Magnification 40×; scale bar: 50 μm. (Panel **A**) Histogram showing the NOS2 mean fluorescence intensity per cell (OD) in control and differently treated cells. * control vs. each experimental condition; ° *Ganostile* vs. other treatments; # Cisplatin vs. any combined treatment; ^ Pt(IV)Ac-POA vs. each combined treatment; + Cisplatin + *Ganostile* vs. other experimental groups.

Double immunolabeling for nitric oxide synthase 2 (NOS2) and actin is reported in Figure 5 in order to evaluate any changes in the free radicals’ level and the possible effect on cell morphology.

After 48hr-CT with *Ganostile* (d–f), a significant reduction in the NOS2 fluorescent signal was observed compared to the control (a–c) conditions, suggesting a reduction in the level of nitrogen radical stress (33.97 ± 3.73 and 21.79 ± 0.85 for Ctrl and *Ganostile*, *respectively*). In the control cells, the actin cytoskeleton (b) was well organized, showing membrane protrusions, and a slight cytoplasmic NOS2 protein labeling (c) was observed. In the samples treated only with *Ganostile*, the cytoskeleton (e) progressively lost the physiological structure and morphology, while NOS2 fluorescence significantly decreased. A cytotoxic effect was detected when the samples were exposed to Cisplatin 40 μM (g–i) and Pt(IV)Ac-POA 10 μM (j–l); in particular, in the samples treated with *Ganostile* + platinum compounds (m–o and p–r for Cisplatin + *Ganostile* and Pt(IV)Ac-POA + *Ganostile*, respectively), NOS2 labeling increased (39.53 ± 1.97, 54.80 ± 4.00, 31.75 ± 1.501 and 48.88 ± 2.14 for Cisplatin, *Ganostile* + Cisplatin, Pt(IV)Ac-POA, and *Ganostile* + Pt(IV)Ac-POA, respectively), distributing itself homogeneously in the cytoplasm (o, r), revealing an increase in the production of reactive species and a reduced functionality of the scavenger molecules present in the cells. In these conditions, the cell volume was extremely reduced and the actin filaments lost their organization (Figure 5, Panel A).

In the context of the redox balance evaluation, superoxide dismutase 2 (SOD2) and cyclooxygenase (COX) enzyme levels (i.e., COX2 and COX4) were also evaluated.

**Figure 6:**
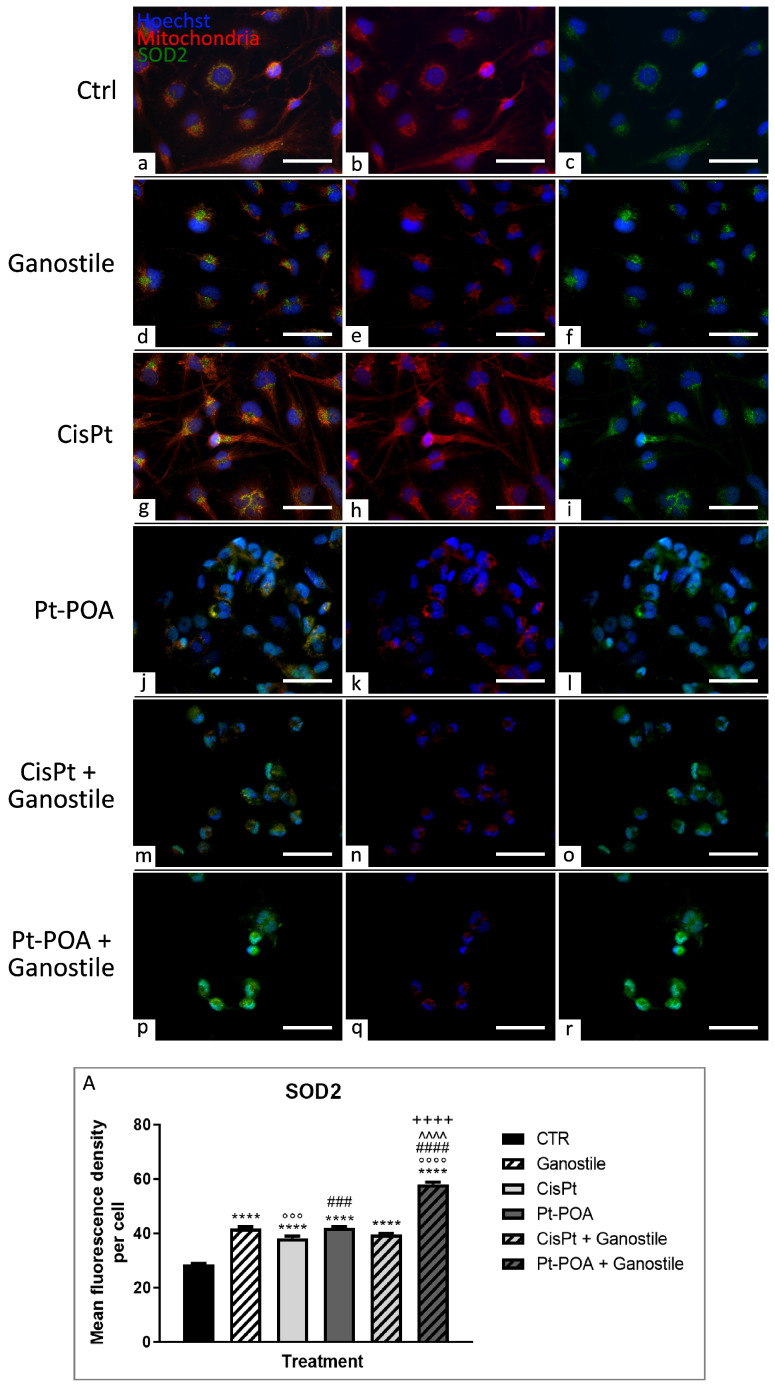
Double immunolabeling for SOD2 (in green) and mitochondria (in red) in controls (**a**–**c**) and differently treated U251 cells, i.e., after 48hr-CT using *Ganostile* 5 mg/mL (**d**–**f**), Cisplatin 40 μM (**g**–**i**), Pt(IV)Ac-POA 10 μM (**j**–**l**), Cisplatin + *Ganostile* (**m**–**o**), or Pt(IV)Ac-POA + *Ganostile* (**p**–**r**). DNA was stained with Hoechst 33258 (blue fluorescence). Magnification 40×; scale bar: 50 μm. (Panel **A**) Histogram showing the SOD2 mean fluorescence intensity per cell (OD) in control and differently treated cells. * control vs. each experimental condition; ° *Ganostile* vs. other treatments; # Cisplatin vs. any combined treatment; ^ Pt(IV)Ac-POA vs. each combined treatment; + Cisplatin + *Ganostile* vs. other experimental groups.

In particular, in the control (a–c) group, SOD2 (Figure 6) appeared expressed at the cytoplasmic level, strongly overlapping with mitochondria (28.51 ± 1.22). Following treatment with *Ganostile* (d–f) supplement, greater homogeneity in the cytosolic distribution of SOD2 (41.84 ± 1.85) and a reduction in mitochondria size were highlighted. Similarly to *Ganostile*-treated cells, after Cisplatin (g–i), Pt(IV)Ac-POA (j–l), or Cisplatin + *Ganostile* (m–o) treatment, an increase in SOD2 immunopositive OD was observed (38.26 ± 2.32, 41.89 ± 1.55 and 39.57 ± 1.30 for Cisplatin, Pt(IV)Ac-POA, and Cisplatin + Ganostile, respectively), partially colocalized with the residual mitochondria. Moreover, in the samples exposed to Pt(IV)Ac-POA + *Ganostile* (p–r), it was possible to note an extremely significant increase in SOD2 immunopositivity (58.00 ± 2.70), strongly detectable also at the nuclear level (Figure 6, Panel A).

**Figure 7:**
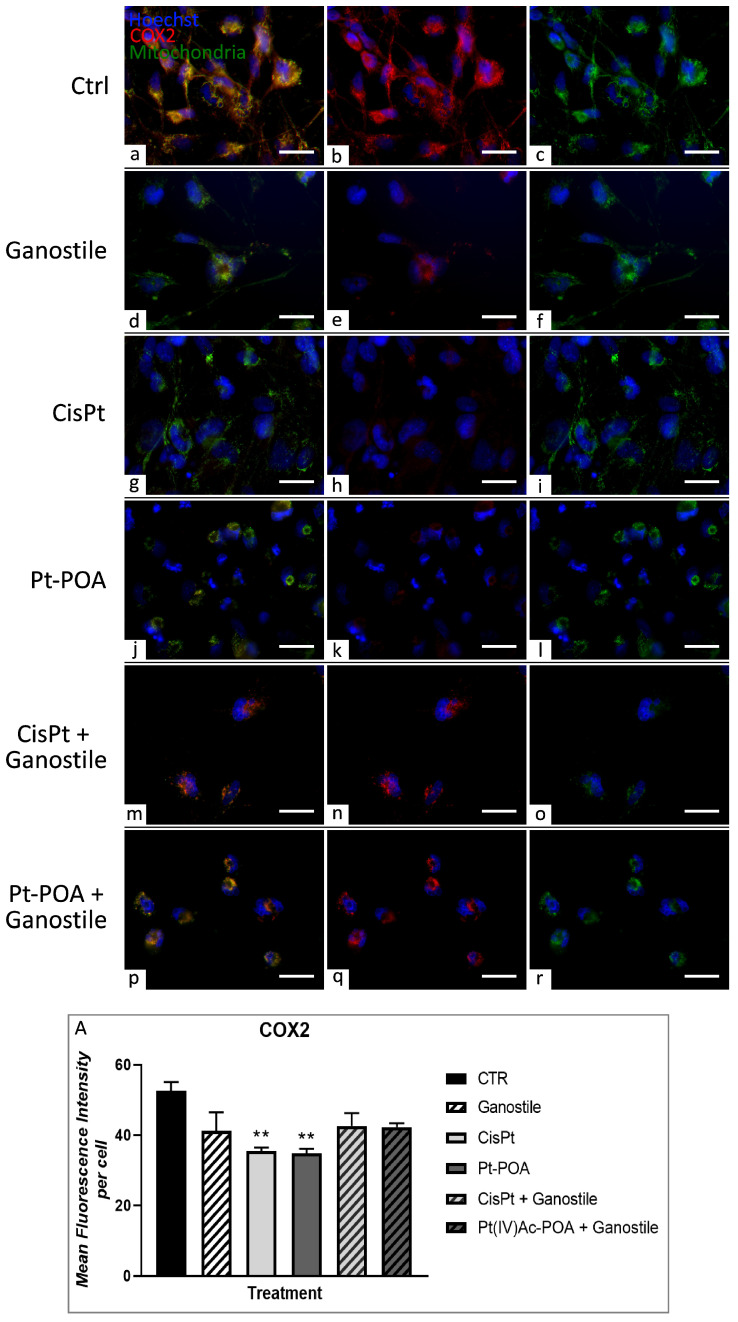
Double immunolabeling for COX2 (in red) and mitochondria (in green) in controls (**a**–**c**) and differently treated U251 cells, i.e., after 48hr-CT using *Ganostile* 5 mg/mL (**d**–**f**), Cisplatin 40 μM (**g**–**i**), Pt(IV)Ac-POA 10 μM (**j**–**l**), Cisplatin + *Ganostile* (**m**–**o**), or Pt(IV)Ac-POA + *Ganostile* (**p**–**r**). DNA was stained with Hoechst 33258 (blue fluorescence). Magnification 60×; scale bar: 25 μm. (Panel **A**) Histogram showing the COX2 mean fluorescence intensity per cell (OD) in control and differently treated cells. * control vs. each experimental condition.

Figure 7 shows the double-immunofluorescent reaction for COX2 and mitochondria. In the control group (a–c), COX2 immunolabeling was clearly detectable in all cells and completely colocalized with mitochondria (52.61 ± 2.50). After *Ganostile* (d–f), a reduction in COX2 OD was observed (41.18 ± 5.37), accompanied by a slight decrease in colocalization with mitochondria, compared to the control. This immunolabeling pattern is maintained even when mycotherapy is used in combination with the two different platinum drugs (m–o and p–r) (42.62 ± 3.64 and 42.32 ± 1.03 for Cispaltin 40 μM + *Ganostile* 5 mg/mL and Pt(IV)Ac-POA 10 μM + *Ganostile*, respectively). However, a very significant reduction in COX2 OD was observed after Cisplatin (g–i) or Pt(IV)Ac-POA (j–l) treatment (35.50 ± 0.99 and 34.89 ± 1.21 for Cisplatin and Pt(IV)Ac-POA, respectively), with a concomitant evident reduction in colocalization with mitochondria, compared to the control group (Figure 7, panel A).

**Figure 8:**
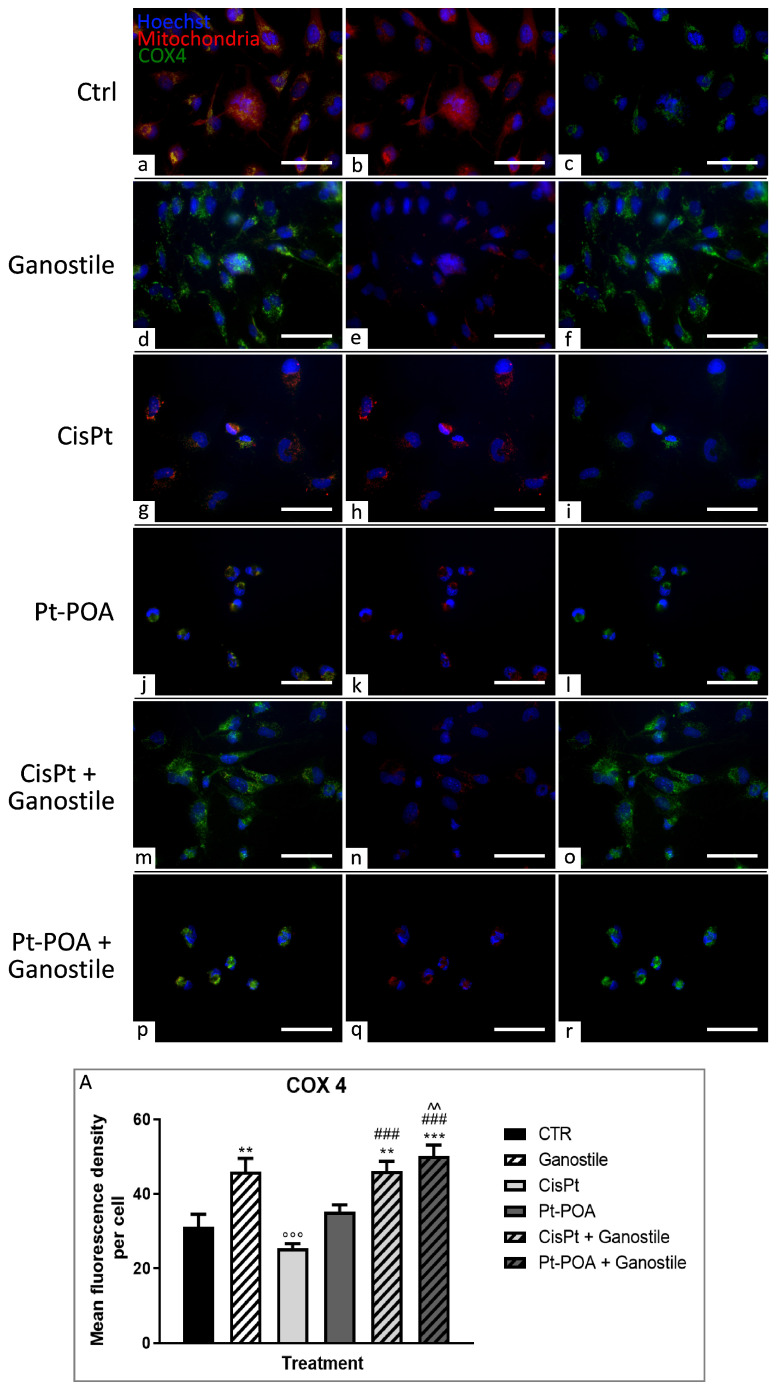
Double immunolabeling for COX4 (in green) and mitochondria (in red) in controls (**a**–**c**) and differently treated U251 cells, i.e., after 48hr-CT using *Ganostile* 5 mg/mL (**d**–**f**), Cisplatin 40 μM (**g**–**i**), Pt(IV)Ac-POA 10 μM (**j**–**l**), Cisplatin + *Ganostile* (**m**–**o**), or Pt(IV)Ac-POA + *Ganostile* (**p**–**r**). DNA was stained with Hoechst 33258 (blue fluorescence). Magnification 40×; scale bar: 50 μm. (Panel **A**) Histogram showing the COX4 mean fluorescence intensity per cell (OD) in control and differently treated cells. * control vs. each experimental condition; # Cisplatin vs. any combined treatment; ^ Pt(IV)Ac-POA vs. each combined treatment. ° *Ganostile* vs. other treatments.

Subsequently, assessing the amount of COX4 enzyme (Figure 8), no differences between the control (a–c) condition and *Ganostile*-treated cells (d–f) were detected in the immunolabeling distribution, but an increase in COX4 immunopositive OD was detected in comparison to the control condition (31.18 ± 3.35). After exposure to Cisplatin 40 μM (g–i) or Pt(IV)Ac-POA 10 μM (j–l), COX4 fluorescence remained almost constant (25.44 ± 1.23 and 35.18 ± 1.85 for Cisplatin and Pt(IV)Ac-POA, respectively). In contrast, after treatment with the combination of platinum compounds with *Ganostile* (m–o and p–r for Cisplatin + *Ganostile* and Pt(IV)Ac-POA + *Ganostile*, respectively), it was observed that there was a statistically significant increase in COX4 immunopositive OD compared to the control (46.11 ± 2.61 and 50.17 ± 2.88, respectively). Moreover, a shrunken and round shape morphology was observed in the Pt(IV)Ac-POA-treated cells; for this reason, mitochondrial and COX4 signals were detectable in the perinuclear cytoplasmatic portion (Figure 8, Panel A).

### 2.4. Changes in Calcium Pathway

**Figure 9:**
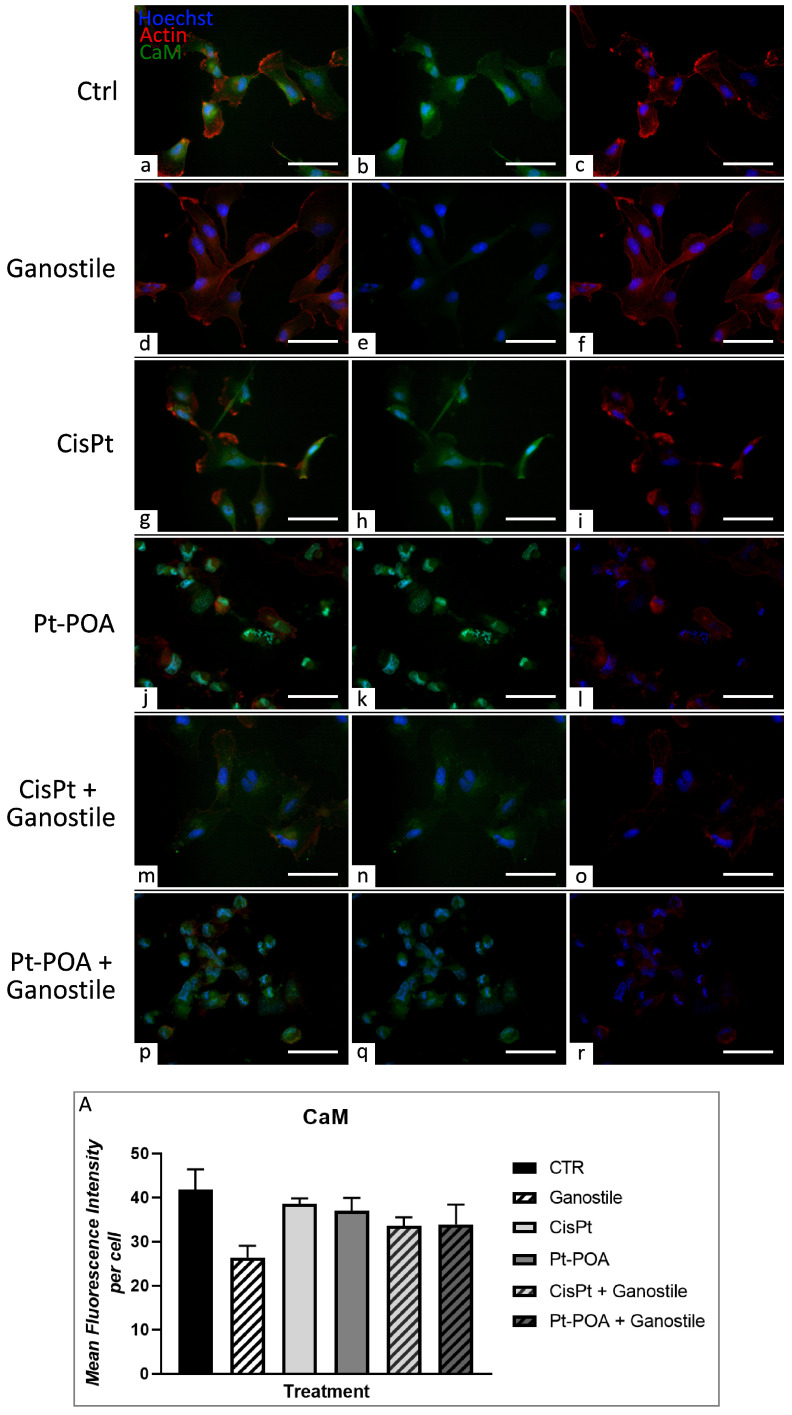
Double immunolabeling for CaM (in green) and actin (in red) in controls (**a**–**c**) and differently treated U251 cells, i.e., after 48hr-CT using *Ganostile* 5 mg/mL (**d**–**f**), Cisplatin 40 μM (**g**–**i**), Pt(IV)Ac-POA 10 μM (**j**–**l**), Cisplatin + *Ganostile* (**m**–**o**), or Pt(IV)Ac-POA + *Ganostile* (**p**–**r**). DNA was stained with Hoechst 33258 (blue fluorescence). Magnification 40×; scale bar: 50 μm. (Panel **A**) Histogram showing the CaM mean fluorescence intensity per cell (OD) in control and differently treated cells.

Possible activation/modification of the calcium pathway was evaluated by analyzing the changes in the expression levels of calmodulin (CaM), one of the main molecules involved in the regulation of cytoplasmatic concentration of calcium, after treatments. Figure 9 reported the double immunofluorescence staining for CaM and actin. In control (a–c) cells, CaM appeared homogeneously distributed in whole cytoplasm. Differently, treatment with Pt(IV)Ac-POA (j–l) leads to an evident agglomeration of CaM immunolabeling at the perinuclear level. Moreover, the quantitative analyses of OD revealed a slight reduction in CaM immunopositivity compared to the control (41.82 ± 4.56), already visible after *Ganostile* (d–f) (26.38 ± 2.66) alone and detectable in all treated groups (g–i, j–l, m–o, and p–r, 38.55 ± 1.25, 36.95 ± 2.93, 33.58 ± 1.95, and 33.88 ± 4.53 for Cisplatin 40 μM, Pt(IV)Ac-POA 10 μM, Cisplatin + *Ganostile*, and Pt(IV)Ac-POA + *Ganostile*, respectively), suggesting a possible modulation of the calcium pathway, already visible after *Ganostile* treatment but evident also after exposure to different platinum compounds, alone or in combination with phyto-mycotherapeutic blend (Figure 9, Panel A).

### 2.5. Apoptotic Pathway Evaluation

**Figure 10:**
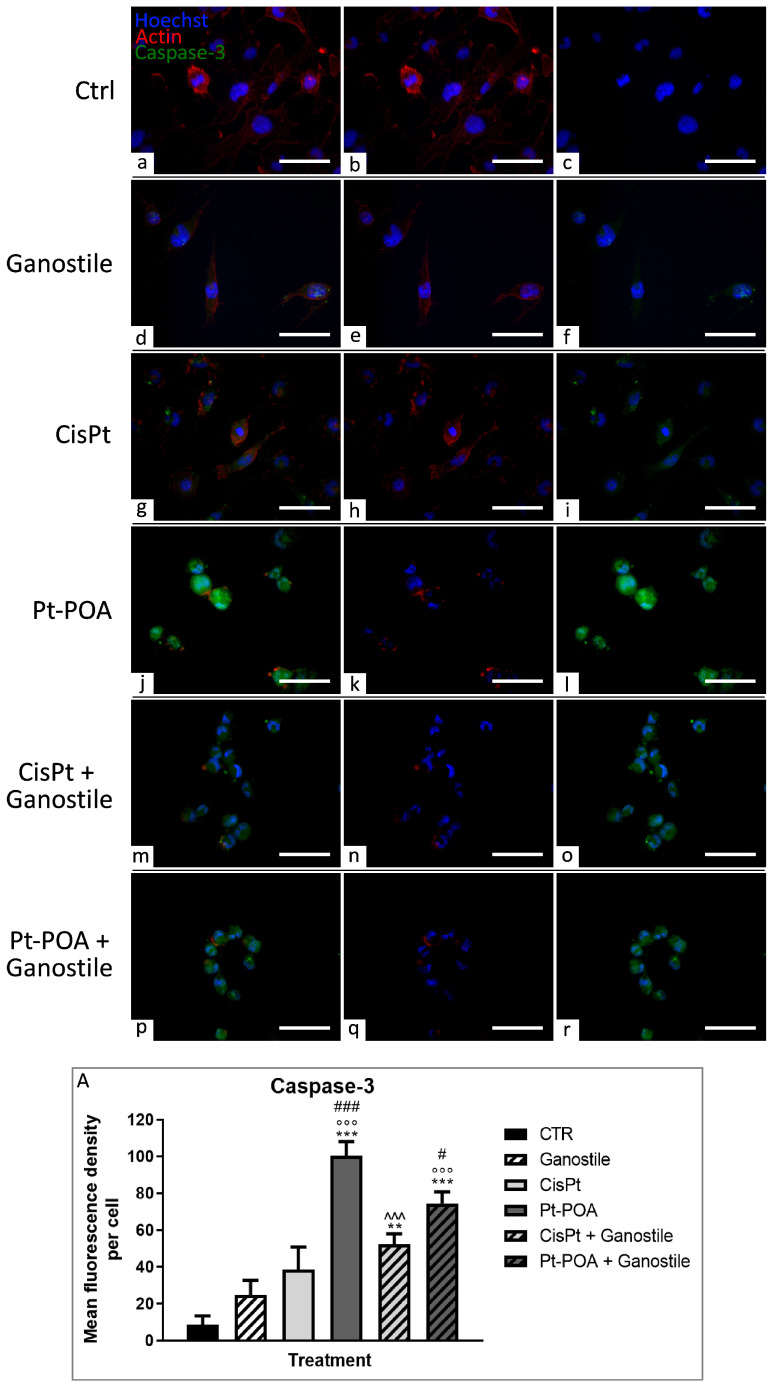
Double immunolabeling for caspase-3 (in green) and actin (in red) in controls (**a**–**c**) and differently treated U251 cells, i.e., after 48hr-CT using *Ganostile* 5 mg/mL (**d**–**f**), Cisplatin 40 μM (**g**–**i**), Pt(IV)Ac-POA 10 μM (**j**–**l**), Cisplatin + *Ganostile* (**m**–**o**), or Pt(IV)Ac-POA + *Ganostile* (**p**–**r**). DNA was stained with Hoechst 33258 (blue fluorescence). Magnification 40×; scale bar: 50 μm. (Panel **A**) Histogram showing the caspase-3 mean fluorescence intensity per cell (OD) in control and differently treated cells. * control vs. each experimental condition; ° *Ganostile* vs. other treatments; # Cisplatin vs. any combined treatment; ^ Pt(IV)Ac-POA vs. each combined treatment.

To question the activation of the apoptotic pathway, the levels of cleaved caspase-3 (Figure 10) were analyzed.

In control (a–c) conditions, as well as after *Ganostile* (d–f), there was a low expression level of caspase-3 (8.55 ± 4.94 and 24.81 ± 7.84 for Ctrl and *Ganostile*, respectively). Of note are the high density of cells and the well-organized actin cytoskeletal structure clearly visible in the control condition. Samples exposed to Cisplatin 40 μM (g–i) showed a slight increase in caspase-3 labeling (38.58 ± 12.25). However, in Pt(IV)Ac-POA 10 μM (j–l) treated cells, an extremely significant increase in caspase-3 immunopositive OD was detected (100.35 ± 7.84), suggesting a strong activation of the apoptotic pathway, also detectable by nucleus fragmentation. Similarly, the use of *Ganostile* in combination with different platinum drugs (m–o and p–r for Cisplatin + *Ganostile* and Pt(IV)Ac-POA + *Ganostile*, respectively) led to a significant increase in the expression levels of cleaved caspase-3 (52.40 ± 5.61 and 74.44 ± 6.46 for Cisplatin + *Ganostile* and Pt(IV)Ac-POA + *Ganostile*, respectively), particularly evident after Pt(IV)Ac-POA + *Ganostile*, where disrupted nuclei, formation of blebs, and apoptotic bodies were easily distinguishable (Figure 10, Panel A).

### 2.6. Ferroptotic Pathway Evaluation

**Figure 11:**
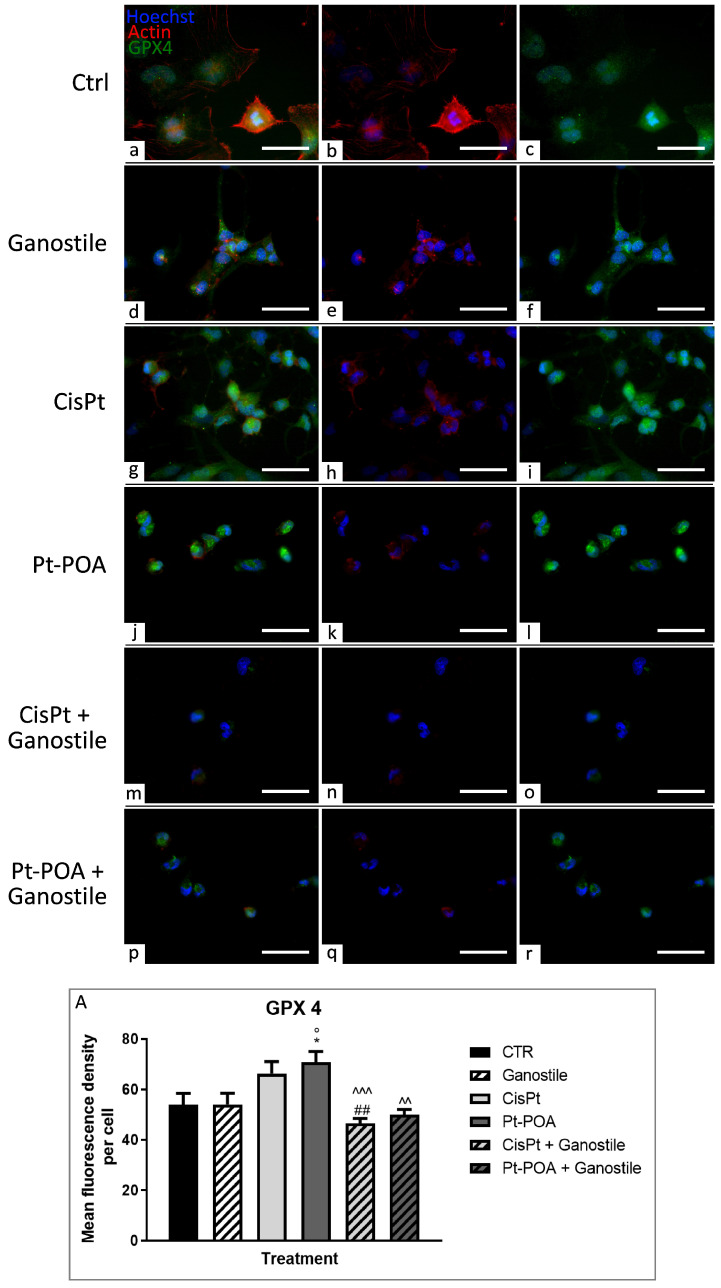
Double immunolabeling for GPX4 (in green) and actin (in red) in controls (**a**–**c**) and differently treated U251 cells, i.e., after 48hr-CT using *Ganostile* 5 mg/mL (**d**–**f**), Cisplatin 40 μM (**g**–**i**), Pt(IV)Ac-POA 10 μM (**j**–**l**), Cisplatin + *Ganostile* (**m**–**o**), or Pt(IV)Ac-POA + *Ganostile* (**p**–**r**). DNA was stained with Hoechst 33258 (blue fluorescence). Magnification 40×; scale bar: 50 μm. (Panel **A**) Histogram showing the GPX4 mean fluorescence intensity per cell (OD) in control and differently treated cells. * control vs. each experimental condition; ° *Ganostile* vs. other treatments; # Cisplatin vs. any combined treatment; ^ Pt(IV)Ac-POA vs. each combined treatment.

To further investigate the link between oxidative stress and cell death pathways, double immunofluorescence labeling for glutathione peroxidase 4 (GPX4), a specific marker of ferroptosis, and actin was performed (Figure 11). Cells incubated with *Ganostile* 5 mg/mL (d–f) showed an intensity of immunofluorescence OD comparable with the control (a–c) (53.98 ± 4.40 *and* 54.02 ± 4.44 for Ctrl and *Ganostile*, respectively), while samples subjected to the only chemotherapeutic agents (g–i and j–l for Cisplatin 40 μM and Pt(IV)Ac-POA 10 μM, respectively) exhibited an increase in GPX OD (66.25 ± 4.77 and 70.73 ± 4.32, for Cisplatin and Pt(IV)Ac-POA, respectively). However, samples undergoing combined therapies of *Ganostile* 5 mg/mL + Cisplatin 40 µM (m–o) or *Ganostile* 5 mg/mL + Pt (IV) Ac-POA 10 µM (p–r) were characterized by the inhibition of the expression of GPX4 in comparison to the only chemical compounds (46.52 ± 1.89 and 49.99 ± 2.04 for *Ganostile* + Cisplatin and *Ganostile* + Pt (IV) Ac-POA, respectively), restoring the basal levels of the marker (Figure 11, Panel A).

### 2.7. Autophagic and Mitophagic Pathway Evaluation

**Figure 12:**
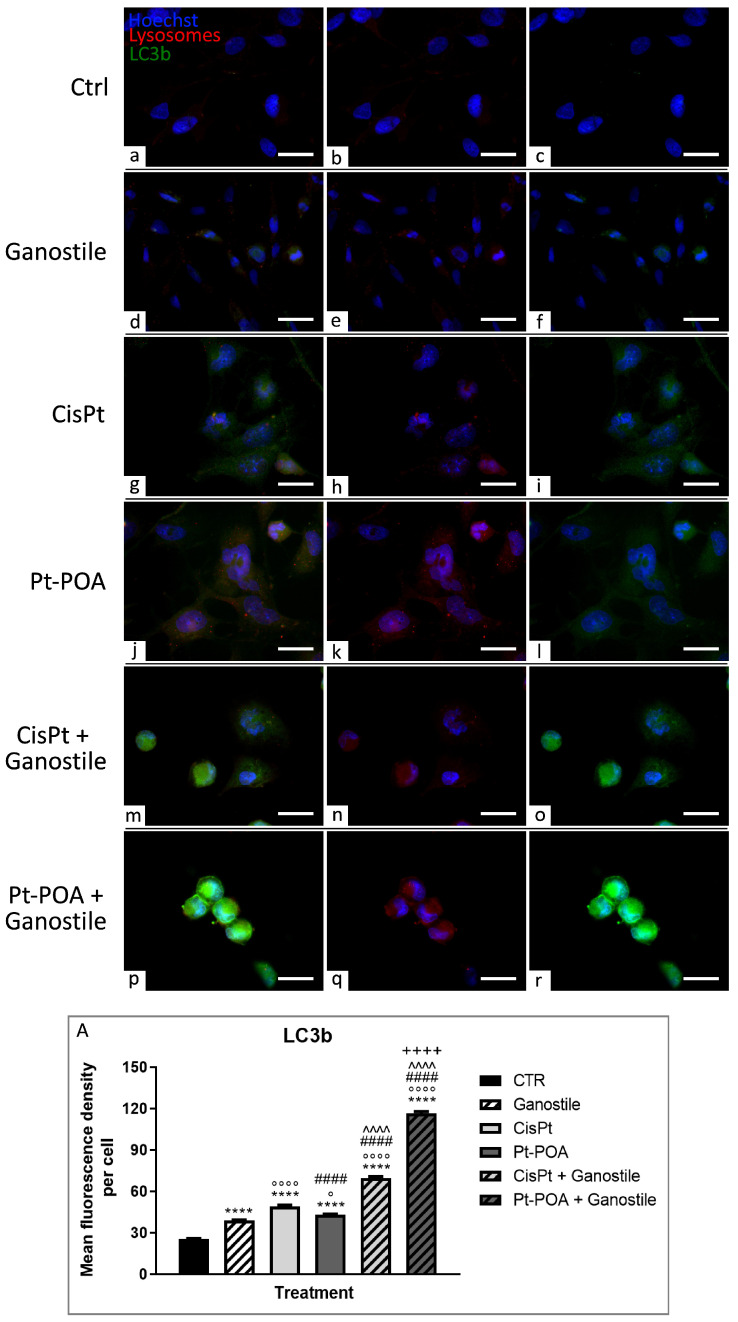
Double immunolabeling for LC3b (in green) and lysosomes (in red) in controls (**a**–**c**) and differently treated U251 cells, i.e., after 48hr-CT using *Ganostile* 5 mg/mL (**d**–**f**), Cisplatin 40 μM (**g**–**i**), Pt(IV)Ac-POA 10 μM (**j**–**l**), Cisplatin + *Ganostile* (**m**–**o**), or Pt(IV)Ac-POA + *Ganostile* (**p**–**r**). DNA was stained with Hoechst 33258 (blue fluorescence). Magnification 60×; scale bar: 25 μm. (Panel **A**) Histogram showing the LC3b mean fluorescence intensity per cell (OD) in control and differently treated cells. * control vs. each experimental condition; ° *Ganostile* vs. other treatments; # Cisplatin vs. any combined treatment; ^ Pt(IV)Ac-POA vs. each combined treatment; + Cisplatin + *Ganostile* vs. other experimental groups.

**Figure 13:**
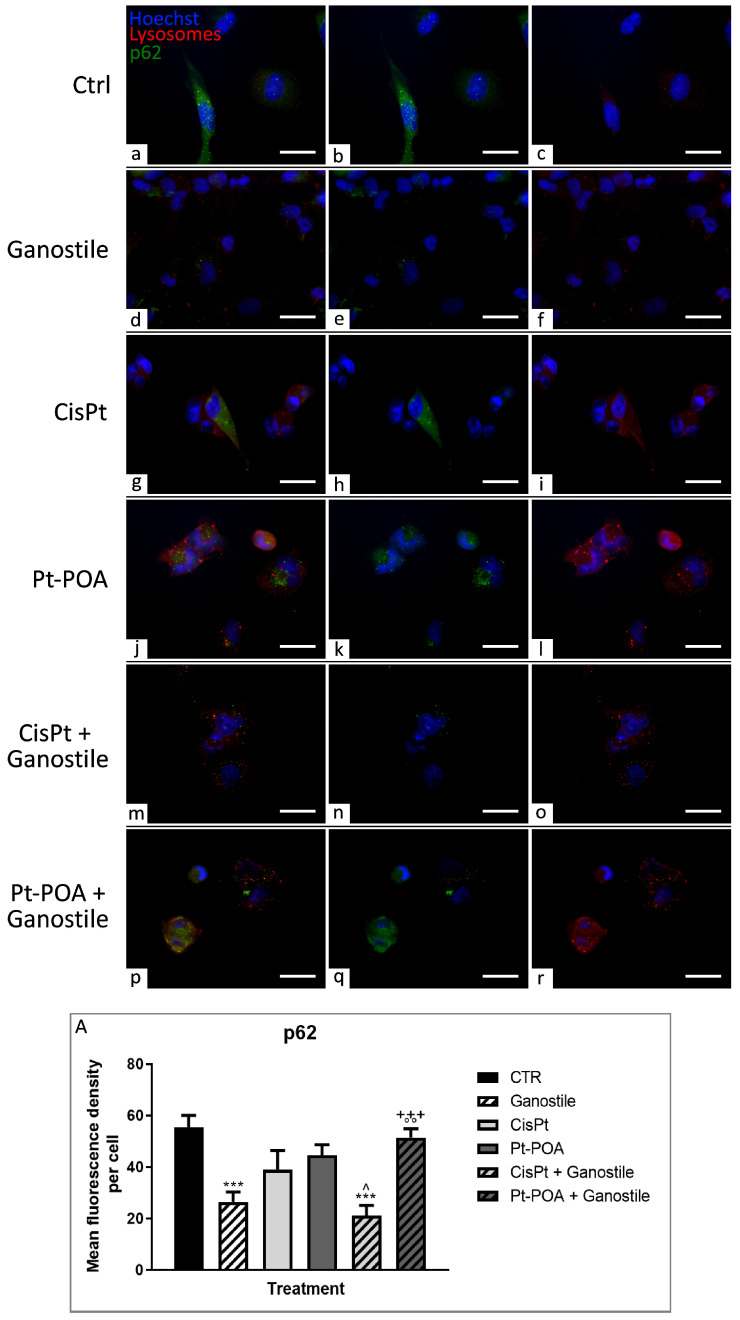
Double immunolabeling for p62 (in green) and lysosomes (in red) in controls (**a**–**c**) and differently treated U251 cells, i.e., after 48hr-CT using *Ganostile* 5 mg/mL (**d**–**f**), Cisplatin 40 μM (**g**–**i**), Pt(IV)Ac-POA 10 μM (**j**–**l**), Cisplatin + *Ganostile* (**m**–**o**), or Pt(IV)Ac-POA + *Ganostile* (**p**–**r**). DNA was stained with Hoechst 33258 (blue fluorescence). Magnification 60×; scale bar: 25 μm. (Panel **A**) Histogram showing the p62 mean fluorescence intensity per cell (OD) in control and differently treated cells. * control vs. each experimental condition; ° *Ganostile* vs. other treatments; ^ Pt(IV)Ac-POA vs. each combined treatment; + Cisplatin + *Ganostile* vs. other experimental groups.

**Figure 14:**
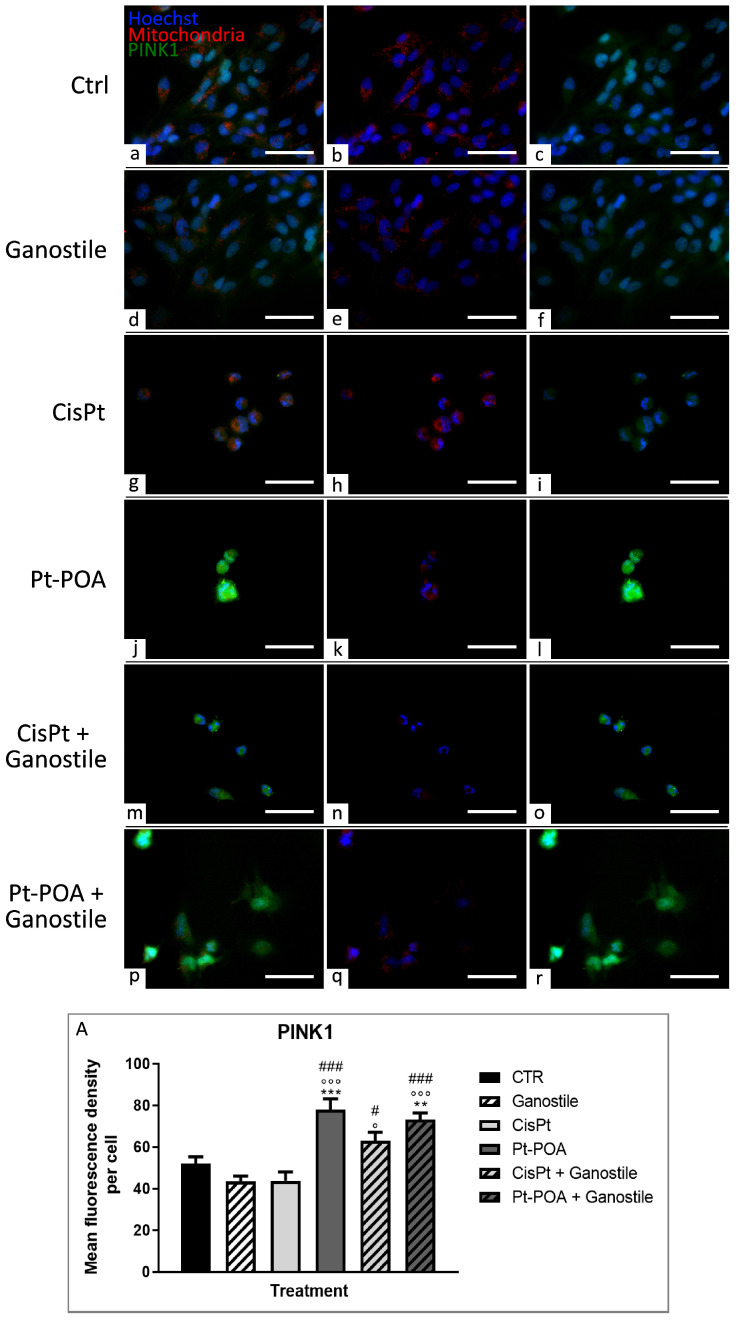
Double immunolabeling for PINK1 (in green) and mitochondria (in red) in controls (**a**–**c**) and differently treated U251 cells, i.e., after 48hr-CT using *Ganostile* 5 mg/mL (**d**–**f**), Cisplatin 40 μM (**g**–**i**), Pt(IV)Ac-POA 10 μM (**j**–**l**), Cisplatin + *Ganostile* (**m**–**o**), or Pt(IV)Ac-POA + *Ganostile* (**p**–**r**). DNA was stained with Hoechst 33258 (blue fluorescence). Magnification 40×; scale bar: 50 μm. (Panel **A**) Histogram showing the PINK1 mean fluorescence intensity per cell (OD) in control and differently treated cells. * control vs. each experimental condition; ° *Ganostile* vs. other treatments; # Cisplatin vs. any combined treatment.

**Figure 15:**
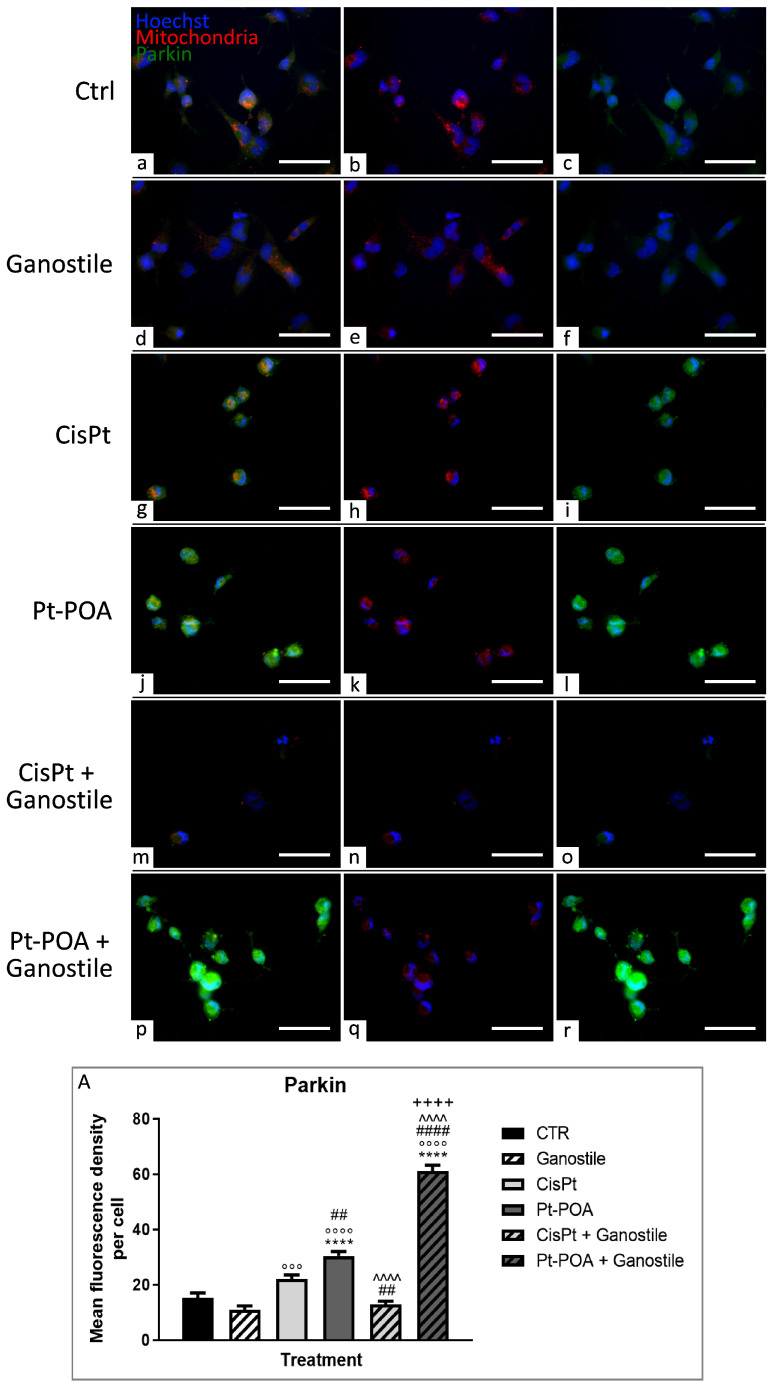
Double immunolabeling for Parkin (in green) and mitochondria (in red) in controls (**a**–**c**) and differently treated U251 cells, i.e., after 48hr-CT using *Ganostile* 5 mg/mL (**d**–**f**), Cisplatin 40 μM (**g**–**i**), Pt(IV)Ac-POA 10 μM (**j**–**l**), Cisplatin + *Ganostile* (**m**–**o**), or Pt(IV)Ac-POA + *Ganostile* (**p**–**r**). DNA was stained with Hoechst 33258 (blue fluorescence). Magnification 40×; scale bar: 50 μm. (Panel **A**) Histogram showing the Parkin mean fluorescence intensity per cell (OD) in control and differently treated cells. * control vs. each experimental condition; ° *Ganostile* vs. other treatments; # Cisplatin vs. any combined treatment; ^ Pt(IV)Ac-POA vs. each combined treatment; + Cisplatin + *Ganostile* vs. other experimental groups.

To evaluate the possible activation of mechanisms required for cellular homeostasis maintenance or other cell death pathways, the expression levels of LC3b (Microtubule-associated protein 1 Light Chain 3b) and p62/SQSTM1 (Sequestosome 1) (Figure 12 and Figure 13, respectively), key players in the autophagic process, were evaluated. Parallelly, immunofluorescence reactions were performed to evaluate possible changes in the expression of PTEN-induced kinase 1 (PINK1) and Parkin, (Figure 14 and Figure 15, respectively), used as specific markers of mitophagy.

In the control (a–c) group, LC3b (Figure 12) protein was sparsely present and homogeneously distributed in the cell cytoplasm (25.25 ± 1.67).

Already, after exposure to *Ganostile* (d–f) supplement, the LC3b protein immunolabeling OD significantly increases (38.99 ± 1.28) and colocalizes itself with lysosome immunofluorescence, indicating an activation of the autophagic process. In cells exposed to platinum-based compounds, Cisplatin 40 μM (g–i) or Pt(IV)Ac-POA 10 μM (j–l), there was a notable rise in the number and size of lysosomes and an increase in the overlap with LC3b immunofluorescence (49.22 ± 2.78 and 42.91 ± SD 2.23 for Cisplatin and Pt(IV)Ac-POA, respectively), a sign of an ongoing autophagosome formation process. When *Ganostile* was combined with platinum compounds (m–o and p–r for Cisplatin + *Ganostile* and Pt(IV)Ac-POA + *Ganostile*, respectively), a strong increase in LC3b immunopositive OD was detected in samples treated with Cisplatin + *Ganostile* (69.84 ± 3.38) and a further rise after Pt(IV)Ac-POA + *Ganostile* (116.43 ± 4.41). Furthermore, LC3b immunolabeling was colocalized with lysosomes, indicating the activation of the process of fusion of the autophagosome with the lysosomes to form the autophagolysosome (Figure 12, Panel A).

As already described for LC3b, the p62/SQSTM1 (Figure 13) protein in control (a–c) cells also had a diffuse distribution throughout the cytoplasm.

Similarly, the lysosomes were also homogeneously distributed and present with modest size and number. In samples treated with the *Ganostile* (d–f), an extremely statistically significant decrease in p62 immunopositive OD was observed (55.42 ± 4.69 and 26.46 ± 3.82 for Ctrl and *Ganostile*, respectively). Following treatment with Cisplatin 40 μM (g–i) or Pt(IV)Ac-POA 10 μM (j–l), the labeling for p62/SQSTM1 was intensely expressed (39.04 ± 7.33 and 44.52 ± 4.16, respectively) and, particularly after Pt(IV)Ac-POA, it colocalized with lysosome fluorescence, which increased their size and concentrated near the nucleus. When the samples were exposed to combined treatment of Cisplatin + *Ganostile* (m–o), a significant reduction in p62 immunofluorescence OD was noted (21.09 ± 4.03). However, after Pt(IV)Ac-POA + *Ganostile* (p–r) treatment, p62 expression levels remained comparable to the control (51.46 ± 3.44) (Figure 13, Panel A).

Immunoreaction for PINK1 is reported in Figure 14. After treatments with *Ganostile* (d–f) and Cisplatin (g–i), PINK1 immunofluorescence OD was comparable to the control (a–c) (43.41 ± 2.60, 43.81 ± 4.15, *and* 51.88 ± 3.44 for *Ganostile*, Cisplatin, and Ctrl, respectively). Only a slight increase in PINK1 OD was detected after Cisplatin + *Ganostile* (m–o) treatment (62.933 ± 4.151). The most significant effect was observed after exposure to Pt(IV)Ac-POA 10 μM (j–l) and its respective combined treatment with the supplement, i.e., Pt(IV)Ac-POA + *Ganostile* (p–r) (73.11 ± 3.25) (Figure 14, Panel A).

Concerning Parkin (Figure 15), immunopositive OD changes between different treatments were in line with the previous trend reported for PINK1 (15.36 ± 1.73, 10.95 ± 1.44, 22.13 ± 1.41, and 12.90 ± 1.16, for Ctrl, *Ganostile*, Cisplatin, and Cisplatin + *Ganostile*).

In particular, a significant increase in Parkin immunofluorescence OD was detected after Pt(IV)Ac-POA (j–l) or Pt(IV)Ac-POA + *Ganostile* (p–r) treatment (30.42 ± 1.68 and 61.14 ± 2.11, respectively) compared to other experimental groups (a–c, d–f, g–i, and m–o for control, *Ganostile*, Cisplatin 40 μM, and Cisplatin + *Ganostile*, respectively) (Figure 15, Panel A).

### 2.8. Ganostile Effects on Cytoplasmic Organelles

**Figure 16:**
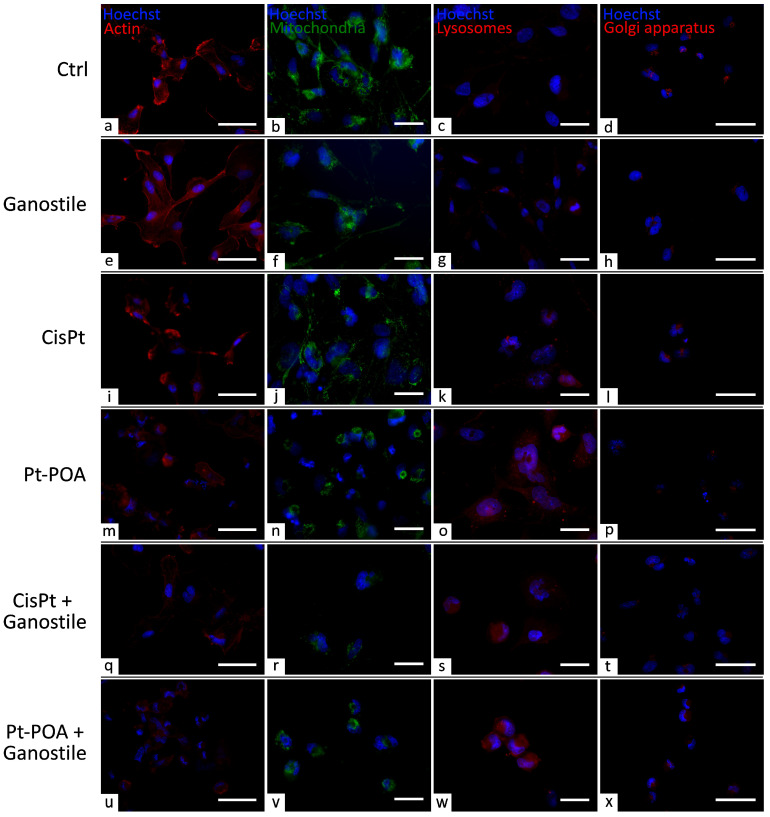
Immunolabeling for actin (**a**,**e**,**i**,**m**,**q**, and **u**, in red), mitochondria (**b**,**f**,**j**,**n**,**r**, and **v**, in green), lysosomes (**c**,**g**,**k**,**o**,**s**, and **w**, in red) and Golgi apparatus (**d**,**h**,**l**,**p**,**t**, and **x**, in red) in controls (**a**–**d**) and differently treated U251 cells, i.e., after 48hr-CT using *Ganostile* 5 mg/mL (**e**–**h**), Cisplatin 40 μM (**i**–**l**), Pt(IV)Ac-POA 10 μM (**m**–**p**), Cisplatin + *Ganostile* (**q**–**t**), or Pt(IV)Ac-POA + *Ganostile* (**u**–**x**). DNA was stained with Hoechst 33258 (blue fluorescence). Magnification 40×; scale bar: 50 μm (**a**,**d**,**e**,**h**,**i**,**l**,**m**,**p**,**q**,**t**,**u**, and **x**); 60×; scale bar: 25 μm (**b**,**c**,**f**,**g**,**j**,**k**,**n**,**o**,**r**,**s**,**v**, and **w**).

**Figure 17:**
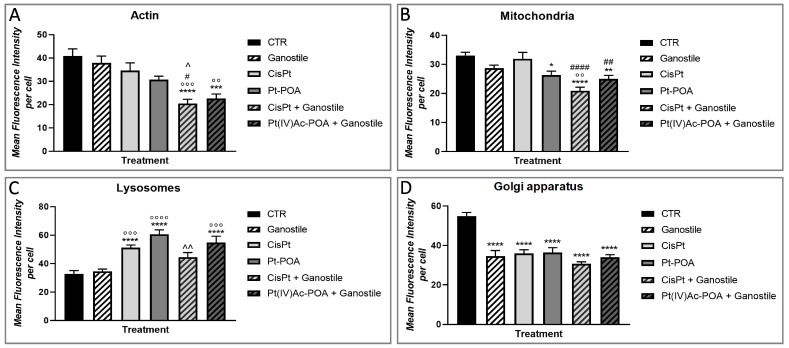
Histogram showing the (Panel **A**) actin, (Panel **B**) mitochondria, (Panel **C**) lysosomes, and (Panel **D**) Golgi apparatus mean fluorescence intensity per cell (OD) in control and differently treated cells. * control vs. each experimental condition; ° *Ganostile* vs. other treatments; # Cisplatin vs. any combined treatment; ^ Pt(IV)Ac-POA vs. each combined treatment.

To identify a possible alteration/modification of cytoplasmic organelles (Figure 16 and Figure 17), typically involved in intracellular metabolism, immunolabeling for actin (a, e, I, m, q, and u), mitochondria (b, f, j, n, r, and v), lysosomes (c, g, k, o, s, and w), and Golgi apparatus (d, h, l, p, t, and x) were performed.

Immunoreaction for actin showed a well-organized cytoskeleton structure in the control group (a). However, already after *Ganostile* (e) (37.81 ± 2.98), a slight decrease in actin immunopositive OD was observed compared to control (40.82 ± 3.09) cells. This immunolabeling reduction is detectable also after exposure to Cisplatin (i) and Pt(IV)Ac-POA (m) (34.57 ± 3.36 and 30.77 ± 1.42, respectively), accompanied by a reorganization of the cellular cytoskeleton, clearly recognizable by the finely localized immunolabeling detected in the cytoplasm of these differently treated groups. Moreover, a significative depletion of actin immunolabeling OD was highlighted after Cisplatin + *Ganostile* (q) or Pt (IV) Ac-POA 10 µM + *Ganostile* (u) (20.44 ± 1.82 and 22.61 ± 1.90, respectively) compared to the control (Figure 16 and Figure 17, Panel A).

Parallelly, the immunofluorescence reaction for mitochondria showed a physiological morphology and widespread distribution of these organelles in the cytoplasm of control (b) cells. After *Ganostile* (f), a slight reduction in mitochondria immunopositive OD was observed compared to the control (28.67 ± 1.06 and 33.01 ± 1.15 for *Ganostile* and control, respectively). No significant difference was detected when comparing Cisplatin (j) (31.88 ± 2.20) and control groups. However, already after Pt(IV)Ac-POA (n) (26.30 ± 1.35), a significant reduction in immunopositive OD was highlighted. This reduction is even more enhanced when comparing the control group with both Cisplatin + *Ganostile* (r) and Pt(IV)Ac-POA + *Ganostile* (v) (20.84 ± 1.28 and 24.97 ± 1.20, respectively) treated cells. Furthermore, only after Pt(IV)Ac-POA or Pt(IV)Ac-POA + *Ganostile* was a cytoplasmatic clusterization of mitochondria well detectable, accompanied by a complete lack of mitochondrial network (Figure 16 and Figure 17, Panel B).

Immunocytochemistry for lysosomes revealed the presence of several vesicles in the cytoplasm of control (c) cells, detectable as well-defined spot fluorescence. Similarly to the control, a weak immunopositive lysosome OD was observed after *Ganostile* (g) (32.85 ± 2.24 and 34.51 ± 1.63 for control and *Ganostile*, respectively). However, a significant increase in immunolabeling OD was detected in Cisplatin (k) (51.09 ± 2.04) or Cisplatin + *Ganostile* (o) (44.49 ± 3.25) groups compared to the control, with a further increase after Pt(IV)Ac-POA (s) (60.51 ± 3.19) or Pt(IV)Ac-POA + *Ganostile* (w) (54.68 ± 4.55) treatment (Figure 16 and Figure 17, Panel C). Subsequently, the immunofluorescence analyses of the Golgi apparatus highlighted the presence of well-preserved organelles in the control (d) group. However, an extremely statistically significant decrease in immunopositive Golgi OD and a concomitant less defined and more disorganized cytoplasmic organelle were detected in all treated experimental groups, i.e., *Ganostile* (**h**), Cisplatin (**i**), Pt(IV)Ac-POA (p), Cisplatin + *Ganostile* (**t**), and Pt(IV)Ac-POA + *Ganostile* (**x**), compared to the control (34.69 ± 2.73, 36.04 ± 1.79, 36.47 ± 2.42, 30.69 ± 1.02, 34.01 ± 1.30, and 54.86 ± 1.84 for *Ganostile*, Cisplatin, Pt (IV) Ac-POA, *Ganostile* + Cisplatin, *Ganostile* + Pt (IV) Ac-POA, and ctrl, respectively). (Figure 16 and Figure 17, Panel D).

### 2.9. Ultrastructural Observation of Morphological Changes

**Figure 18:**
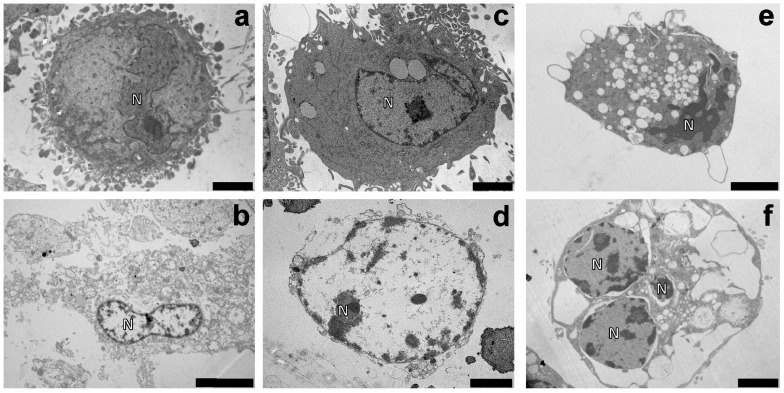
TEM ultrastructural analysis of control (**a**) and *Ganostile* 5 mg/mL (**b**), Cisplatin 40 μM (**c**), Cisplatin + *Ganostile* (**d**), Pt(IV)Ac-POA 10 μM (**e**), and Pt(IV)Ac-POA + *Ganostile* (**f**) treated cells. N: nucleus. Scale bar; 2 μm (**a**,**c**–**f**); 5 μm (**b**).

Figure 18 shows reported images obtained by electron transmission microscopy showing a comparison between control (a) and treated (b–f) groups. In control cells, the nucleus and organelles in the cytoplasm were detectable and well organized. In contrast, *Ganostile* (b) treated cells showed a typical visual pattern of ferroptosis; the cell showed a reduced cytoplasm and a decreased number of mitochondria, with disappearance of mitochondrial cristae and rupture of the outer mitochondrial membrane. Furthermore, even if the nucleus and the nuclear envelope were still visible, the electron-dense structures of nuclear bodies were no longer appreciable.

In cells treated for 48hr with Cisplatin (c), the nucleus was still intact and the plasma membrane was also distinguishable but the cytoplasm presented large vacuoles, typical features of the autophagic process.

Ultrastructural alterations, markers of autophagic pathway activation, are clearly detectable also after 48hr-CT with Pt(IV)Ac-POA at 10 μM (e), characterized by (i) a strong alteration in the morphology of the nucleus and chromatin condensation, (ii) cytoplasm engulfed by numerous vacuoles containing degrading material, including mitochondria, and (iii) disorganized endoplasmic reticulum and Golgi apparatus.

However, cells treated using *Ganostile* and platinum compounds (d and f for Cisplatin + *Ganostile* and Pt(IV)Ac-POA + *Ganostile*, respectively) showed evident necroptotic features. Indeed, disagreement of the plasma membrane, enlargement of the organelles, chromatin condensation to the nuclear lamina, disappearance of the nucleosome, and dilatation of the perinuclear space were detected.

## 3. Discussion

Glioblastoma (GBM) is the most common and malignant of the primary tumors of the central nervous system and the most aggressive of gliomas. GBM often induces relapsing forms and it is characterized by acquired chemo- and radio-resistance. Here, we have investigated the potentiality of combining different treatments that could affect the tumor cells through multiple targets, thus creating a synergic antitumor effect. The cells were exposed to 48hr of continuous treatment with platinum-based chemotherapeutic agents and myco-phytotherapeutic supplement; therefore, the acute cytotoxic effects exerted by the compounds were evaluated, through in vitro assays, immunofluorescence techniques, and ultrastructural analysis. The results obtained thanks to the synergistic pharmacological action of the tested compounds seem promising; all the samples subjected to combined treatments showed a higher mortality rate and a lower proliferation rate than the samples subjected to the different treatments administered individually. In particular, a reduction in cell density compared to the control was noted not only between the samples treated with *Ganostile* alone but also between the samples with combined treatment. These data were also confirmed by an increase in cleaved caspase-3 cell positivity and a reduction in PCNA immunolabeling in samples subjected to platinum compounds combined with the phytotherapy supplement, in line with previous literature data evaluating the synergistic effects of naturally derived proteins and chemotherapy in the regulation of cell death mechanisms [24]. Similarly, an alteration in cell morphology with impaired actin cytoskeletal organization is already visible after treatment with *Ganostile* alone. This cytoskeletal alteration worsens significantly when this mycotherapy is used in combination with our two different platinum compounds, resulting in a consequent collapse of the microfilaments and a progressive nuclear fragmentation and cytoplasmatic organelle reorganization, typical aspects of the apoptotic process. The idea of an activated cell death mechanism is also corroborated by the strong alteration in Golgi observed already after *Ganostile* treatment, which became more evident in both *Ganostile* + Cisplatin and *Ganostile* + Pt(IV)Ac-POA treated cells. Moreover, mitochondria, another key cellular organelle whose dysfunction plays a crucial role in survival, metabolism, proliferation, and cell death of glioblastoma, are found to be deeply compromised after *Ganostile* + Cisplatin and *Ganostile* + Pt(IV)Ac-POA treatments, possibly resulting in mitophagy and mitochondrial apoptosis pathways activation [25]. Parallelly, the activation of cell death pathways is confirmed by the increase in lysosome expression levels, clearly observable after the use of the fourth-generation platinum compound, alone or in combination with our mycotherapeutic blend.

This finding was also supported by an increase in the activation of the autophagic pathway, made visible thanks to labeling for SQSTM1/p62 and LC3b proteins, typically involved in this process [26,27]. These data correlated also with PINK1 and Parkin, which, being involved in the mitophagic process, play tumor-relevant suppressor functions [28]. After treatment with Pt(IV)Ac-POA and the combination of the supplement and the fourth-generation platinum compound, an increased immunofluorescence was observed for both markers. Additionally, if, in control conditions, Parkin was mostly localized in the cytosol, after the combined treatments, it translocated from the cytosol to the mitochondria, probably responding to PINK1 accumulation on the outer mitochondrial membrane due to mitochondrial depolarization, thus activating intracellular pathways to clear the damaged mitochondria [29,30,31].

A possible explanation for the mitochondrial dysfunction was evaluated through the study of markers of oxidative stress, SOD2 and NOS2. An initial increase in the activation of antioxidant barriers was noted in the samples treated with *Ganostile*; in particular, the expression of these enzymes seemed significantly increased after combined administration of the platinum compounds used with the myco-phytotherapeutic supplement, probably indicating an intensification of the state of oxidative stress and cellular impairment. Moreover, a tendency of SOD2 to lose its cytoplasmic distribution and to overlap with the nuclei, particularly in the samples treated with *Ganostile* + Pt(IV)Ac-POA, emerged, demonstrating the failure of antioxidant and antiapoptotic defenses. Similarly, in the sample subjected to treatments with Cisplatin 40 μM or Pt(IV)Ac-POA 10 μM and *Ganostile*, NOS2 labeling increased, distributing itself homogeneously in the cytoplasm, revealing a compensatory activation in response to the rise in the production of reactive species and reduced functionality of the scavenger molecules present in the cells [32]. Furthermore, from the analysis of COX4 amount, a rising level of the enzyme was given by the supplementation of *Ganostile* with chemotherapeutic agents, indicating increased reactive oxygen species formation [33,34,35]. Consistently, a reduced amount of GPX4 was highlighted in samples receiving combined treatment, suggesting an increased sensitivity to ferroptosis and oxidative stress [36]. Parallelly, our results demonstrated a reduction in COX2 expression levels, already detectable after *Ganostile* and maintained following platinum compounds. This enzyme is a typical marker of carcinogenesis, overexpressed in GBM and strongly linked with tumor progression as well as the resistance of cancer cells to conventional pharmacological and radiological treatments. The reduced expression of this tumor-promoting factor corroborates the antitumoral effects linked to the possible synergism between *Ganostile* and platinum compounds [23,37,38,39]. Moreover, *Ganostile* alone is able to reduce intracellular levels of calmodulin and, in synergism with our different platinum drugs, may help to modify/alter the calcium pathway in GBM cell line. CaM is a ubiquitous regulator protein involved in several cellular pathways, starting from actin cytoskeleton reorganization to intercellular communication and Ca^2+^ homeostasis; this latter mechanism is known to be a key player in physiological and pathophysiological conditions in both neuron and glial cells. Our results are in line with previous literature data, demonstrating how several cytoplasmatic proteins involved in the maintenance of intracellular calcium levels play important roles in the regulation of different aspects of glioblastoma and how high cytoplasmatic levels of the multifunctional calcium-binding protein calmodulin are a typical feature of GBM not detectable in low-grade glial tumors [40].

An important alteration in the redox balance dictated by the use of the supplement was confirmed by observations in transmission electron microscopy. In fact, if the samples subjected to chemotherapeutic drugs alone mainly showed signs of death via autophagy, those subjected to mycotherapy or combined treatment showed signs correlated with a form of regulated necrotic cell death, such as ferroptosis and necroptosis. Therefore, if the platinum-compound alone seems to trigger autophagy and apoptosis, the combined effects of chemotherapeutic drug + *Ganostile* induced ferroptotic and necroptotic pathway activation. Overall, we hypothesize that the combined use of Pt(IV)Ac-POA + *Ganostile* could therefore induce synergic effects on U251 cell line, improving the antiproliferative/cytotoxic effect mediated by chemotherapeutic treatment alone. While the role of the autophagic pathway is still debated, as the boundary between resistance to oncotherapy-induced damage and activation of an effective cell death mechanism seems thin, recent studies highlighted the fundamental role of ferroptotic and necroptotic signaling in cancer therapy. Indeed, the induction of nonapoptotic regulated cell death emerged as a new efficient strategy not only for the induction of death in cancer cells but also for the promotion of antitumor immunity [41,42].

In the current investigation, the potential efficacy of *Ganostile*, a phyto-mycotherapeutic preparation composed of four diverse extracts, and its synergistic effect with conventional chemotherapeutic drugs were evaluated with the aim of assessing the usefulness of the blend as a valuable adjuvant in cancer treatment. Among the extracts composing *Ganostile*, *G. lucidum*, well known for its anticancer activity, due to its immunomodulatory properties and direct cytotoxic action on tumor cell lines [43,44,45,46,47,48,49], has been already extensively investigated in gliomas and glioblastoma models [13,50,51,52]. Similarly, new potential multitarget effects of *A. membranaceus* on glioma cells have been reported in recent years [53,54,55]. Diversely, a paucity of data exists concerning the effects induced by the other two extracts composing the blend, i.e., *E. senticosus* and *E. purpurea*, on glioma or GBM cell lines. Nevertheless, the immunomodulatory action and antioxidant properties of both *E. senticosus* and *E. purpurea* have been recorded [56,57,58,59,60,61], revealing their ability to activate an ancillary onco-suppression mechanism and to reduce the side effects of chemotherapy [62]. Therefore, although the current data are preliminary and further investigations could be carried out to better highlight possible synergistic effects of the proposed treatments, evaluating potential in vitro antitumor activity after combined treatments using the proposed chemotherapeutic and phyto-mycotherapeutic treatments (e.g., through the use of Chou and Talalay or similar formalism), as well as the possible in vivo metabolism, immunomodulatory effects, and blood–brain barrier permeability, the present investigation seems to support the use of this phyto-micotherapeutic preparation as a promising adjuvant in the field of integrative oncology, also paving the way to further in-depth studies devoted to clarifying the cellular/molecular mechanisms induced by each natural extract composing *Ganostile*.

## 4. Materials and Methods

### 4.1. Cell Lines

Human U251 MG cell line (Sigma-Aldrich, Rome, Italy) was cultured in Eagle’s minimal essential medium (EMEM) supplemented with 2 mM L-glutamine, 1% nonessential amino acids (NEAA), 1% sodium pyruvate, 10% fetal bovine serum (FBS), and 1% penicillin/streptomycin. To control the effects of the treatments also on nontumoral cells, primary culture of normal human fibroblasts (experimental protocols approved by the Ethical Committee of the IRCCS Policlinico San Donato [63]) was cultivated in Dulbecco’s Modified Eagle Medium (D-MEM) containing 15% FBS, 2 mM of glutamine, and 1% penicillin/streptomycin. Both cell lines were maintained at 37 °C in a humidified atmosphere (95% air/5% CO_2_). Entirely cell culture reagents were acquired from Celbio s.p.a. and Euroclone s.p.a. (Pero, Milan, Italy).

### 4.2. Ganostile Composition and Treatments

A myco-phytotherapeutic supplement based on *G. lucidum*, called *Ganostile* (Miconet s.r.l, Pavia, Italy), was used to treat U251 cell line and fibroblast. The supplement composition (Table 1) is ganoderma (*Ganoderma lucidum* (Curtis) P.Karst., fungus) dry extract, echinacea (*Echinacea purpurea* (L.) Moench., root) dry extract, eleutherococcus (*Eleuterococcus senticosus* (Rupr et Maxim) Maxim., root) dry extract, astragalus (*Astragalus membranaceus* Moench., Root) dry extract, bulking agent: calcium phosphate, anti-caking agent: silicon dioxide, and magnesium salts of fatty acids.

**Figure 19:**
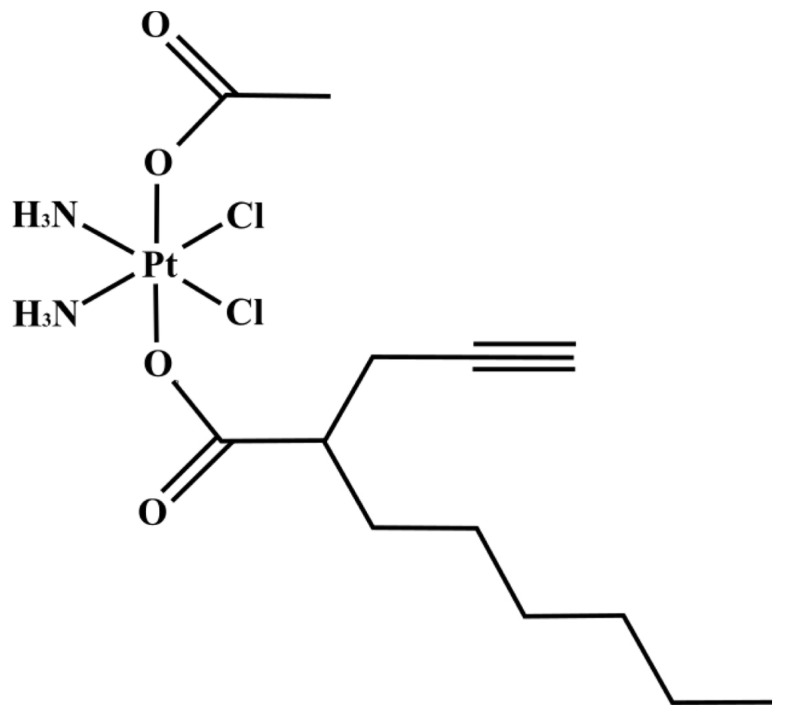
Chemical structure of Pt(IV)Ac-POA.

The cells were treated with Cisplatin (Teva Pharma, Milan, Italy) at a concentration of 40 μM and with the prodrug Pt(IV)Ac-POA (synthesized and provided by the Department of Sciences and Technological Innovation (DiSIT), University of Piemonte Orientale) (Figure 19) at a concentration of 10 μM. These concentrations were chosen based on previous in vitro investigations [64,65]. To evaluate the adjuvant effect of *G. lucidum* with chemotherapeutic agents, the treatments were combined as follows: Cisplatin 40 μM + *Ganostile* 5 mg/mL and Pt(IV)Ac-POA 10 μM + *Ganostile* 5 mg/mL. All the reagents used have been solubilized in the culture medium.

Cell lines were exposed to the natural supplement according to the standard acute test, which consists of 48hr of continuous treatment (48hr-CT).

### 4.3. Ganostile Dose Selection: Cell Viability/Proliferation Evaluated by MTS Assay

Taking into consideration the maximum daily dose recommended for human oral supplementation (about 1 g/day), with the aim of identifying the proper *Ganostile* dose to be used in the subsequent determinations, as a first experimental step, a range of *Ganostile* concentrations was evaluated through the MTS [3-(4,5-dimethylthiazol-2-yl)-5-(3-carboxymethoxyphenyl)-2-(4-sulfophenyl)-2H-tetrazolium] assay both on U251 and primary culture of human fibroblasts. MTS assay, as a cell viability test, was performed using the CellTiter 96^®^ AQueous One Solution Cell Proliferation Assay (Promega, Milan, Italy) kit. A volume of 200 μL of cells was suspended at a density of 10,000 cells/well and transferred to a 96-well plate and incubated at 37 °C for 24 h, in a humidified atmosphere containing 5% of CO_2_. Afterward, the culture medium was replaced for 48hr with a medium containing several concentrations of the supplement (100, 50, 25, 10, 5, 2.5, 1, 0.5, 0.25, and 0 mg/mL). Each condition was replicated at least 4 times, to which 4 specific “whites” were associated with the absorbance normalization. After the incubation, the culture medium was replaced with fresh medium and, to each well, 20 μL of MTS solution, previously brought to room temperature (RT), was added. Subsequently, the plates were incubated for 3 hr at 37 °C and read at 490 nm, using the ELx808TM Absorbance Microplate Reader (Bio-Tek Instruments, Inc., Winooski, VT, USA) plate reader. This protocol was executed in triplicate to obtain statistical data and the percent cell viability was calculated using the following formula: Cell viability (%) = (Abs490 treated cells/Abs490 control cells) × 100.

### 4.4. Flow Cytometry

Control and treated cells were cultured in 75 cm^2^ flasks and, after being subjected to 48hr-CT of treatments, were washed in sterile phosphate-buffered saline (PBS), detached by trypsinization, and filtered with 40 µm cell strainers. The cells were then permeabilized in 70% ethanol for 10 min, treated with RNase A 100 U/mL, and stained for 10 min at RT with 50 μg/mL propidium iodide (PI) (Sigma-Aldrich, Milan, Italy) 1 h. The preparation was processed with a Partec PAS III flow cytometer (Partec GmbH, Münster, Germany) and PI red fluorescence was detected with a 610 nm long-pass emission filter. Data were analyzed with Flowing Software (version 2.5.1).

### 4.5. Immunocytochemical Reactions for Fluorescence Microscopy

The procedure was performed as previously described [64]. Briefly, control and treated cells were grown on coverslips for 48hr and, after being subjected to 48hr-CT of treatments, they were fixed with 4% formalin for 20 min and post-fixed with 70% ethanol at −20 °C for at least 24 hr. Samples were rehydrated for 10 min in PBS and then incubated for 1 hr with primary antibodies diluted in PBS (Table 2) at RT in a dark moist chamber. After 3 washes in PBS of 5 min each, coverslips were exposed for 45 min to secondary antibodies diluted in PBS (1:200, Alexa Fluor, Molecular Probes, Invitrogen, San Diego, CA). At the end of the incubation and after other washing in PBS, sections were counterstained for DNA with 0.1 µg mL^−1^ Hoechst 33258 (Sigma-Aldrich, Milano, Italy), washed with PBS, and mounted in a drop of Mowiol (Calbiochem, Inalco, Italy) for fluorescence microscopy analysis.

An Olympus BX51 microscope equipped with a 100 W mercury lamp was used under the following conditions: 330–385 nm excitation filter (excf), 400 nm dichroic mirror (dm), and 420 nm barrier filter (bf) for Hoechst 33258; 450–480 nm excf, 500 nm dm, and 515 nm bf for the fluorescence of Alexa 488; 540 nm excf, 580 nm dm, and 620 nm bf for Alexa 594. Images were recorded with an Olympus MagnaFire camera system and processed with the Olympus Cell F software (version 3.1). Time exposure during acquisition was maintained constant for all the experimental groups to make fluorescence intensity comparable between different experimental conditions. The fluorescence intensity was subsequently analyzed using ImageJ software 1.51 (NIH, Bethesda, MD, USA).

### 4.6. Transmission Electron Microscopy (TEM)

Control and treated cells were cultured in 75 cm^2^ flasks and, after being subjected to 48hr-CT of treatments, were washed in PBS, detached by mild trypsinization (0.25% trypsin in PBS containing 0.05% EDTA) and collected by centrifugation at 800 rpm for 5 min. The samples were fixed for 2 h at RT with 2.5% glutaraldehyde (Polysciences, Inc., Warrington, PA, USA) in culture medium and centrifuged and washed several times with PBS. Samples were post-fixed in 1% OsO4 (Sigma Chemical Co., St. Louis, MO, USA) for 2 h at RT and washed in distilled water. The cell pellets were pre-embedded in 2% agar and dehydrated with increasing concentrations of acetone (30, 50, 70, 90, and 100%). Finally, the pellets were embedded in epoxy resin EM-bed812 (Electron Microscopy Sciences, Hatfield, PA, USA) and polymerized at 60 °C for 48hr. Sections of 70–80 nm were cut on a Reichert OM-U3 ultramicrotome and collected on nickel grids and counterstained with uranyl acetate for 10 min and with lead citrate for 3 min. Sections were observed under a JEM 1200 EX II (JEOL, Peabody, MA, USA) electron microscope operating at 100 kV and equipped with a MegaView G2 CCD camera (Olympus OSIS, Tokyo, Japan).

### 4.7. Statistical Analysis

Each experiment was carried out as three independent replicates. The values obtained were expressed as mean ± standard deviation (SD) in the MTS viability assay or mean ± standard error of the mean (SEM) for other analyses. The D’Agostino & Pearson, Anderson–Darling, Kolmogorov–Smirnov, and Shapiro–Wilk tests were performed to evaluate the normality distributions of parameters. Subsequently, data were analyzed to verify statistically significant differences. For data that did not pass the normality test, the analysis was conducted using the Kruskal–Wallis test followed by Dunn’s test for multiple comparisons. Diversely, for normally distributed results, the analysis was performed employing one-way ANOVA followed by Bonferroni’s post hoc test. The differences were considered statistically significant for *p* < 0.05 (*), *p* < 0.01 (**), *p* < 0.001 (***), and *p* < 0.0001 (****). For immunocytochemical evaluations, statistical significance was expressed as follows: * control vs. each experimental condition; ° *Ganostile* vs. other treatments; # Cisplatin vs. any combined treatment; ^ Pt(IV)Ac-POA vs. each combined treatment; and + Cisplatin + *Ganostile* vs. other experimental groups. In the histograms, each bar indicates any statistical significance derived from the comparison with the preceding experimental groups. Differences were analyzed for statistical significance using GraphPad Prism 8.0 (GraphPad Software Inc., San Diego, CA, USA).

## 5. Conclusions

In conclusion, when administered alone, *Ganostile* did not reach levels of efficacy that could be considered a full-fledged drug. Instead, used as an adjuvant, in combination with Cisplatin and Pt(IV)Ac-POA, it has proved effective in its antiproliferative and antitumoral actions. Indeed, it increased the effects of the compounds, supplied individually, demonstrating a good synergistic action.

Further studies will be necessary to evaluate in detail the effect of the different components of the supplement, both on in vivo and in vitro tumor models, e.g., potential in vivo immunomodulatory effects of our phyto-mycotherapeutic supplementation and in vitro anticancer effects using different tumor cell lines (T98G and B50), in order to deepen their possible role in improving current clinical practice in the treatment of glioblastoma.

## Figures and Tables

**Table 1:** Details on *Ganostile* supplement composition.

Medicinal Mushroom/Plant	*Ganostile* Content
Ganoderma lucidum	27%
Eleuterococcus senticosus	27%
Echinacea purpurea	33%
Astragalus membranaceus	13%

**Table 2:** Primary antibodies used for immunofluorescence reactions.

Antigen	Primary Antibody	Dilution
*Caspase-3*	Rabbit monoclonal anti-Cleaved caspase-3 (Cell Signaling Technology, Danvers, MA, USA)	1:200
*PCNA*	Mouse monoclonal (PC10) anti-PCNA (Abcam, Cambridge, MA, USA)	1:200
*Mitochondria*	Human autoimmune serum anti-mitochondrial isoform of HSP70 [66]	1:200
*Golgi*	Rabbit polyclonal anti-GRP94 (Abcam, Cambridge, MA, USA) [67]	1:200
*Lysosomes*	Human autoimmune serum anti-lysosomal proteinase [66]	1:400
*LC3B*	Rabbit polyclonal anti-LC3B (Cell Signaling Technology, Danvers, MA, USA)	1:400
*p62/SQSTM1*	Mouse monoclonal anti- p62/SQSTM1 (Abcam, Cambridge, MA, USA)	1:100
*Parkin*	Mouse monoclonal anti-Parkin (Abcam, Cambridge, MA, USA)	1:500
*PINK1*	Rabbit polyclonal anti-PINK1 (Abcam, Cambridge, MA, USA)	1:500
*NOS2*	Mouse monoclonal anti-NOS2 (C-11) (Santa Cruz Biotechnology, Dallas, TX, USA)	1:200
*COX2*	Mouse polyclonal anti-COX2 (M-19) (Santa Cruz Biotechnology, Dallas, TX, USA)	1:200
*COX4*	Mouse monoclonal (20E8C12) anti-COX4 (Abcam, Cambridge, MA, USA)	1:500
*α-tubulin*	Mouse monoclonal anti-α-tubulin (Cell Signaling Technology, Danvers, MA, USA)	1:1000
*Actin*	Alexa 488-Phalloidin/Alexa 594-Phalloidin (Molecular Probes, Invitrogen)	1:500

## Data Availability

The data presented in this study are available in the article.

## References

[1] Stupp R., Mason W.P., van den Bent M.J., Weller M., Fisher B., Taphoorn M.J.B., Belanger K., Brandes A.A., Marosi C., Bogdahn U. (2005). Radiotherapy plus Concomitant and Adjuvant Temozolomide for Glioblastoma. N. Engl. J. Med..

[2] Rossi P., Difrancia R., Quagliariello V., Savino E., Tralongo P., Randazzo C.L., Berretta M. (2018). B-Glucans from Grifola Frondosa and Ganoderma Lucidum in Breast Cancer: An Example of Complementary and Integrative Medicine. Oncotarget.

[3] Qu L., Li S., Zhuo Y., Chen J., Qin X., Guo G. (2017). Anticancer Effect of Triterpenes from Ganoderma Lucidum in Human Prostate Cancer Cells. Oncol. Lett..

[4] Jiang D., Wang L., Zhao T., Zhang Z., Zhang R., Jin J., Cai Y., Wang F. (2017). Restoration of the Tumor-Suppressor Function to Mutant P53 by Ganoderma Lucidum Polysaccharides in Colorectal Cancer Cells. Oncol. Rep..

[5] Ferreira I.C.F.R., Heleno S.A., Reis F.S., Stojkovic D., Queiroz M.J.R.P., Vasconcelos M.H., Sokovic M. (2015). Chemical Features of Ganoderma Polysaccharides with Antioxidant, Antitumor and Antimicrobial Activities. Phytochemistry.

[6] Du G.H., Wang H.X., Yan Z., Liu L.Y., Chen R.Y. (2017). Anti-Tumor Target Prediction and Activity Verification of Ganoderma Lucidum Triterpenoids. Zhongguo Zhongyao Zazhi.

[7] Sohretoglu D., Huang S. (2018). Ganoderma Lucidum Polysaccharides as An Anti-Cancer Agent. Anticancer Agents Med. Chem..

[8] Xu Z., Chen X., Zhong Z., Chen L., Wang Y. (2011). Ganoderma Lucidum Polysaccharides: Immunomodulation and Potential Anti-Tumor Activities. Am. J. Chin. Med..

[9] Wang J., Yuan Y., Yue T. (2014). Immunostimulatory Activities of β-d-Glucan from Ganoderma Lucidum. Carbohydr. Polym..

[10] Wu G.S., Guo J.J., Bao J.L., Li X.W., Chen X.P., Lu J.J., Wang Y.T. (2013). Anti-Cancer Properties of Triterpenoids Isolated from Ganoderma Lucidum—A Review. Expert Opin. Investig. Drugs.

[11] Barbieri A., Quagliariello V., Del Vecchio V., Falco M., Luciano A., Amruthraj N.J., Nasti G., Ottaiano A., Berretta M., Iaffaioli R.V. (2017). Anticancer and Anti-Inflammatory Properties of Ganoderma Lucidum Extract Effects on Melanoma and Triple-Negative Breast Cancer Treatment. Nutrients.

[12] Martínez-Montemayor M.M., Ling T., Suárez-Arroyo I.J., Ortiz-Soto G., Santiago-Negrón C.L., Lacourt-Ventura M.Y., Valentín-Acevedo A., Lang W.H., Rivas F. (2019). Identification of Biologically Active Ganoderma Lucidum Compounds and Synthesis of Improved Derivatives That Confer Anti-Cancer Activities in Vitro. Front. Pharmacol..

[13] Cheng A.Y., Chien Y.C., Lee H.C., Hsieh Y.H., Yu Y.L. (2020). Water-Extracted Ganoderma Lucidum Induces Apoptosis and S-Phase Arrest via Cyclin-CDK2 Pathway in Glioblastoma Cells. Molecules.

[14] Chiu H.F., Fu H.Y., Lu Y.Y., Han Y.C., Shen Y.C., Venkatakrishnan K., Golovinskaia O., Wang C.K. (2017). Triterpenoids and Polysaccharide Peptides-Enriched Ganoderma Lucidum: A Randomized, Double-Blind Placebo-Controlled Crossover Study of Its Antioxidation and Hepatoprotective Efficacy in Healthy Volunteers. Pharm. Biol..

[15] Liu Y.Q., Wang X.L., He D.H., Cheng Y.X. (2021). Protection against Chemotherapy- and Radiotherapy-Induced Side Effects: A Review Based on the Mechanisms and Therapeutic Opportunities of Phytochemicals. Phytomedicine.

[16] Roda E., De Luca F., Di Iorio C., Ratto D., Siciliani S., Ferrari B., Cobelli F., Borsci G., Priori E.C., Chinosi S. (2020). Novel Medicinal Mushroom Blend as a Promising Supplement in Integrative Oncology: A Multi-Tiered Study Using 4t1 Triple-Negative Mouse Breast Cancer Model. Int. J. Mol. Sci..

[17] Wang R., Shi L., Liu S., Liu Z., Song F., Sun Z., Liu Z. (2019). Mass Spectrometry-Based Urinary Metabolomics for the Investigation on the Mechanism of Action of Eleutherococcus Senticosus (Rupr. & Maxim.)Maxim. Leaves against Ischemic Stroke in Rats. J. Ethnopharmacol..

[18] Wang Y.H., Meng Y., Zhai C., Wang M., Avula B., Yuk J., Smith K.M., Isaac G., Khan I.A. (2019). The Chemical Characterization of Eleutherococcus Senticosus and Ci-Wu-Jia Tea Using UHPLC-UV-QTOF/MS. Int. J. Mol. Sci..

[19] Hou R., Xu T., Li Q., Yang F., Wang C., Huang T., Hao Z. (2020). Polysaccharide from Echinacea Purpurea Reduce the Oxidant Stress in Vitro and in Vivo. Int. J. Biol. Macromol..

[20] Auyeung K.K., Han Q.-B., Ko J.K. (2016). Astragalus Membranaceus: A Review of Its Protection Against Inflammation and Gastrointestinal Cancers. Am. J. Chin. Med..

[21] Rangone B., Ferrari B., Astesana V., Masiello I., Veneroni P., Zanellato I., Osella D., Bottone M.G. (2018). A New Platinum-Based Prodrug Candidate: Its Anticancer Effects in B50 Neuroblastoma Rat Cells. Life Sci..

[22] Ferrari B., Urselli F., Gilodi M., Camuso S., Priori E.C., Rangone B., Ravera M., Veneroni P., Zanellato I., Roda E. (2020). New Platinum-Based Prodrug Pt(IV)Ac-POA: Antitumour Effects in Rat C6 Glioblastoma Cells. Neurotox. Res..

[23] Ferrari B., Roda E., Priori E.C., De Luca F., Facoetti A., Ravera M., Brandalise F., Locatelli C.A., Rossi P., Bottone M.G. (2021). A New Platinum-Based Prodrug Candidate for Chemotherapy and Its Synergistic Effect With Hadrontherapy: Novel Strategy to Treat Glioblastoma. Front. Neurosci..

[24] Hsin I.L., Wang S.C., Li J.R., Ciou T.C., Wu C.H., Wu H.M., Ko J.L. (2016). Immunomodulatory Proteins FIP-Gts and Chloroquine Induce Caspase-Independent Cell Death via Autophagy for Resensitizing Cisplatin-Resistant Urothelial Cancer Cells. Phytomedicine.

[25] Li W., Xu X. (2023). Advances in Mitophagy and Mitochondrial Apoptosis Pathway-Related Drugs in Glioblastoma Treatment. Front. Pharmacol..

[26] Kumar A.V., Mills J., Lapierre L.R. (2022). Selective Autophagy Receptor P62/SQSTM1, a Pivotal Player in Stress and Aging. Front. Cell Dev. Biol..

[27] Lee Y.K., Lee J.A. (2016). Role of the Mammalian ATG8/LC3 Family in Autophagy: Differential and Compensatory Roles in the Spatiotemporal Regulation of Autophagy. BMB Rep..

[28] Poole L.P., Macleod K.F. (2021). Mitophagy in Tumorigenesis and Metastasis. Cell. Mol. Life Sci..

[29] Agnihotri S., Golbourn B., Huang X., Remke M., Younger S., Cairns R.A., Chalil A., Smith C.A., Krumholtz S.-L., Mackenzie D. (2022). Correction: PINK1 Is a Negative Regulator of Growth and the Warburg Effect in Glioblastoma. Cancer Res..

[30] Chen J., Zhou C., Yi J., Sun J., Xie B., Zhang Z., Wang Q., Chen G., Jin S., Hou J. (2021). Metformin and Arsenic Trioxide Synergize to Trigger Parkin/Pink1-Dependent Mitophagic Cell Death in Human Cervical Cancer HeLa Cells. J. Cancer.

[31] Kumar R., Reichert A.S. (2021). Common Principles and Specific Mechanisms of Mitophagy from Yeast to Humans. Int. J. Mol. Sci..

[32] Papi S., Ahmadizar F., Hasanvand A. (2019). The Role of Nitric Oxide in Inflammation and Oxidative Stress. Immunopathol. Persa.

[33] Chang B., Guan H., Wang X., Chen Z., Zhu W., Wei X., Li S. (2021). Cox4i2 Triggers an Increase in Reactive Oxygen Species, Leading to Ferroptosis and Apoptosis in HHV7 Infected Schwann Cells. Front. Mol. Biosci..

[34] Oliva C.R., Markert T., Yancey Gillespie G., Griguer C.E. (2015). Nuclear-Encoded Cytochrome c Oxidase Subunit 4 Regulates BMI1 Expression and Determines Proliferative Capacity of High-Grade Gliomas. Oncotarget.

[35] Onishi K., Miyake M., Tatsumi Y., Hori S., Nakai Y., Onishi S., Iemura Y., Owari T., Itami Y., Iida K. (2020). Inhibitory Effect of Orally Administered 5-Aminolevulinic Acid on Prostate Carcinogenesis in the FVB-Transgenic Adenocarcinoma of a Mouse Prostate (FVB-TRAMP) Model. Asian Pac. J. Cancer Prev..

[36] Seibt T.M., Proneth B., Conrad M. (2019). Role of GPX4 in Ferroptosis and Its Pharmacological Implication. Free Radic. Biol. Med..

[37] Li J., Hu X., Luo T., Lu Y., Feng Y., Zhang H., Liu D., Fan X., Wang Y., Jiang L. (2021). N-2-(Phenylamino) Benzamide Derivatives as Novel Anti-Glioblastoma Agents: Synthesis and Biological Evaluation. Eur. J. Med. Chem..

[38] Qiu J., Shi Z., Jiang J. (2017). Cyclooxygenase-2 in Glioblastoma Multiforme. Drug Discov. Today.

[39] Oksuz E., Gorgisen G., Ozdemir H., Gulacar I.M., Oto G. (2021). Effect of Paracetamol in the Proliferation of Glioblastoma Cell Line: The Role of Apoptosis, COX-2 and Cyclin B Expressions. Turk. Neurosurg..

[40] Azab M.A., Alomari A., Azzam A.Y. (2020). Featuring How Calcium Channels and Calmodulin Affect Glioblastoma Behavior. A Review Article. Cancer Treat. Res. Commun..

[41] Tang R., Xu J., Zhang B., Liu J., Liang C., Hua J., Meng Q., Yu X., Shi S. (2020). Ferroptosis, Necroptosis, and Pyroptosis in Anticancer Immunity. J. Hematol. Oncol..

[42] Gao W., Wang X., Zhou Y., Wang X., Yu Y. (2022). Autophagy, Ferroptosis, Pyroptosis, and Necroptosis in Tumor Immunotherapy. Signal Transduct. Target. Ther..

[43] Shahid A., Chen M., Yeung S., Parsa C., Orlando R., Huang Y. (2023). The Medicinal Mushroom Ganoderma Lucidum Prevents Lung Tumorigenesis Induced by Tobacco Smoke Carcinogens. Front. Pharmacol..

[44] Gao X., Homayoonfal M. (2023). Exploring the Anti-Cancer Potential of Ganoderma Lucidum Polysaccharides (GLPs) and Their Versatile Role in Enhancing Drug Delivery Systems: A Multifaceted Approach to Combat Cancer. Cancer Cell Int..

[45] Ye T., Ge Y., Jiang X., Song H., Peng C., Liu B. (2023). A Review of Anti-Tumour Effects of Ganoderma Lucidum in Gastrointestinal Cancer. Chin. Med..

[46] Gariboldi M.B., Marras E., Ferrario N., Vivona V., Prini P., Vignati F., Perletti G. (2023). Anti-Cancer Potential of Edible/Medicinal Mushrooms in Breast Cancer. Int. J. Mol. Sci..

[47] Rahimnia R., Akbari M.R., Yasseri A.F., Taheri D., Mirzaei A., Ghajar H.A., Farashah P.D., Baghdadabad L.Z., Aghamir S.M.K. (2023). The Effect of Ganoderma Lucidum Polysaccharide Extract on Sensitizing Prostate Cancer Cells to Flutamide and Docetaxel: An in Vitro Study. Sci. Rep..

[48] Liu L., Yu Z., Chen J., Liu B., Wu C., Li Y., Xu J., Li P. (2023). Lucialdehyde B Suppresses Proliferation and Induces Mitochondria-Dependent Apoptosis in Nasopharyngeal Carcinoma CNE2 Cells. Pharm. Biol..

[49] Wu X., Wu Q., Wang Y., Liu Y., Li Z., Liu Q., Huang Z., Li M., Zhang B., Zhan Q. (2023). Aqueous-Soluble Components of Sporoderm-Removed Ganoderma Lucidum Spore Powder Promote Ferroptosis in Oral Squamous Cell Carcinoma. Chin. J. Cancer Res..

[50] Li R., Tang X., Xu C., Guo Y., Qi L., Li S., Ren Q., Jie W., Chen D. (2021). Circular RNA NF1-419 Inhibits Proliferation and Induces Apoptosis by Regulating Lipid Metabolism in Astroglioma Cells. Recent Pat. Anticancer Drug Discov..

[51] Wang C., Shi S., Chen Q., Lin S., Wang R., Wang S., Chen C. (2018). Antitumor and Immunomodulatory Activities of Ganoderma Lucidum Polysaccharides in Glioma-Bearing Rats. Integr. Cancer Ther..

[52] Wang C., Lin D., Chen Q., Lin S., Shi S., Chen C. (2018). Polysaccharide Peptide Isolated from Grass-Cultured Ganoderma Lucidum Induces Anti-Proliferative and pro-Apoptotic Effects in the Human U251 Glioma Cell Line. Oncol. Lett..

[53] Sun J.Y., Yang H., Miao S., Li J.P., Wang S.W., Zhu M.Z., Xie Y.H., Wang J.B., Liu Z., Yang Q. (2009). Suppressive Effects of Swainsonine on C6 Glioma Cell in Vitro and in Vivo. Phytomedicine.

[54] Liu Q., Sun Y., Zheng J.M., Yan X.L., Chen H.M., Chen J.K., Huang H.Q. (2015). Formononetin Sensitizes Glioma Cells to Doxorubicin through Preventing EMT via Inhibition of Histone Deacetylase 5. Int. J. Clin. Exp. Pathol..

[55] Feng Y., Zhu P., Wu D., Deng W. (2023). A Network Pharmacology Prediction and Molecular Docking-Based Strategy to Explore the Potential Pharmacological Mechanism of Astragalus Membranaceus for Glioma. Int. J. Mol. Sci..

[56] Panossian A., Seo E.J., Efferth T. (2018). Novel Molecular Mechanisms for the Adaptogenic Effects of Herbal Extracts on Isolated Brain Cells Using Systems Biology. Phytomedicine.

[57] Graczyk F., Gębalski J., Makuch-Kocka A., Gawenda-Kempczyńska D., Ptaszyńska A.A., Grzyb S., Bogucka-Kocka A., Załuski D. (2022). Phenolic Profile, Antioxidant, Anti-Enzymatic and Cytotoxic Activity of the Fruits and Roots of Eleutherococcus Senticosus (Rupr. et Maxim.) Maxim. Molecules.

[58] Panossian A., Seo E.J., Efferth T. (2019). Effects of Anti-Inflammatory and Adaptogenic Herbal Extracts on Gene Expression of Eicosanoids Signaling Pathways in Isolated Brain Cells. Phytomedicine.

[59] Yu T., He Y., Chen H., Lu X., Ni H., Ma Y., Chen Y., Li C., Cao R., Ma L. (2022). Polysaccharide from Echinacea Purpurea Plant Ameliorates Oxidative Stress-Induced Liver Injury by Promoting Parkin-Dependent Autophagy. Phytomedicine.

[60] Han N.R., Park H.J., Ko S.G., Moon P.D. (2023). The Protective Effect of a Functional Food Consisting of Astragalus Membranaceus, Trichosanthes Kirilowii, and Angelica Gigas or Its Active Component Formononetin against Inflammatory Skin Disorders through Suppression of TSLP via MDM2/HIF1α Signaling Pathways. Foods.

[61] Li Z., Qi J., Guo T., Li J. (2023). Research Progress of Astragalus Membranaceus in Treating Peritoneal Metastatic Cancer. J. Ethnopharmacol..

[62] Guo S., Li Y., Su H., Meng M., Xi J., Mo G., Chen X. (2021). Aidi Injection as Adjunctive Treatment to Gemcitabine-Based Chemotherapy for Advanced Non-Small Cell Lung Cancer: A Systematic Review and Meta-Analysis. Pharm. Biol..

[63] Malatesta M., Giagnacovo M., Renna L.V., Cardani R., Meola G., Pellicciari C. (2011). Cultured Myoblasts from Patients Affected by Myotonic Dystrophy Type 2 Exhibit Senescence-Related Features: Ultrastructural Evidence. Eur. J. Histochem..

[64] Bottone M.G., Soldani C., Veneroni P., Avella D., Pisu M., Bernocchi G. (2008). Cell Proliferation, Apoptosis and Mitochondrial Damage in Rat B50 Neuronal Cells after Cisplatin Treatment. Cell Prolif..

[65] Roda E., De Luca F., Priori E.C., Ratto D., Pinelli S., Corradini E., Mozzoni P., Poli D., Mazzini G., Bottone M.G. (2023). The Designer Drug APHP Affected Cell Proliferation and Triggered Deathly Mechanisms in Murine Neural Stem/Progenitor Cells. Biology.

[66] Alpini C., Lotzniker M., Valaperta S., Bottone M.G., Malatesta M., Montanelli A., Merlini G. (2012). Characterization for Anti-Cytoplasmic Antibodies Specificity by Morphological and Molecular Techniques. Autoimmun. Highlights.

[67] Santin G., Piccolini V.M., Veneroni P., Barni S., Bernocchi G., Bottone M.G. (2011). Different Patterns of Apoptosis in Response to Cisplatin in B50 Neuroblastoma Rat Cells. Histol. Histopathol..

